# Genome-Wide Reidentification and Expression Analysis of *MADS-Box* Gene Family in Cucumber

**DOI:** 10.3390/ijms26083800

**Published:** 2025-04-17

**Authors:** Zimo Wang, Jingshu Chang, Jing Han, Mengmeng Yin, Xuehua Wang, Zhonghai Ren, Lina Wang

**Affiliations:** 1 Shandong Collaborative Innovation Center of Fruit & Vegetable Quality and Efficient Production, College of Horticultural Science and Engineering, Shandong Agricultural University, Tai’an 271018, China; 2024010032@sdau.edu.cn (Z.W.); 2022110303@sdau.edu.cn (J.C.); 2021110287@sdau.edu.cn (M.Y.); zhren@sdau.edu.cn (Z.R.); 2 College of Agriculture and Biology, Liaocheng University, Liaocheng 252000, China; hanjing@lcu.edu.cn

**Keywords:** gene reidentification, cucumber, MADS-box, transcription factor

## Abstract

MADS-box transcription factors play a crucial role in plant growth and development. Although previous genome-wide analyses have investigated the MADS-box family in cucumber, this study provides the first comprehensive reannotation of the *MADS-box* gene family in *Cucumis sativus* using updated Cucurbitaceae genome data, offering novel insights into the gene family’s evolution and functional diversity. The results show that a total of 48 *CsMADS-box* genes were identified in the V3 version of cucumber, while 3 of the 43 genes identified in the V1 version were duplicated. The V1 version actually has only 40 genes. Additionally, we analyzed the variability in protein sequences and found that the amino acid sequences of 14 genes showed no differences between the two versions of the database, while the amino acid sequences of 29 genes exhibited significant differences. The further analysis of conserved motifs revealed that although the amino acid lengths of 15 genes had changed, their conserved motifs remained unchanged; however, the conserved motifs of 12 genes had altered. Furthermore we found that motif1 and motif2 were present in most proteins, indicating that they are highly conserved. Gene structure analysis revealed that most type I (Mα, Mβ) *MADS-box* genes lack introns, whereas type II (MIKC) genes exhibit a similar structure with a higher number of introns. Chromosomal localization analysis indicated that *CsMADS-box* genes are unevenly distributed across the seven chromosomes of cucumber. Promoter region analysis showed that the promoter regions of *CsMADS-box* genes contain response elements related to plant growth and development, suggesting that *CsMADS-box* genes may be extensively involved in plant growth and development. Different *CsMADS-box* genes exhibit specific high expression in roots, stems, leaves, tendrils, male flowers, female flowers, and ovaries, suggesting that these genes play crucial roles in the growth, development, reproduction and morphogenesis of cucumber. Moreover, 26, 18, 8, and 10 *CsMADS-box* genes were differentially expressed under high temperature, NaCl and/or silicon, downy mildew, and powdery mildew treatments, respectively. Interestingly, *CsMADS07* and *CsMADS16* responded to all tested stress conditions. These findings provide a reference and basis for further investigation into the function and mechanisms of the *MADS-box* genes for resistance breeding in cucumber.

## 1. Introduction

The MADS-box gene family is one of the most widely studied transcription factor genes in plants. It is characterized by a highly conserved DNA-binding MADS domain at the N-terminus, containing 56–60 amino acids [1,2]. Plant *MADS-box* genes can be divided into two categories according to their evolutionary lineages: type I and type II. Type II genes are also known as MIKC genes because they share a common structure of four domains. In addition to the MADS (M) domain, MIKC-type genes also contain three other conserved domains: I, K and C domains [3]. The I domain is responsible for the specificity of DNA binding dimer formation, the K domain is involved in protein-protein interaction, and the C domain is involved in transcriptional activation [4,5,6]. Based on its structural characteristics, MIKC can be further divided into two types: MIKC* and MIKC^C^ [7]. Compared with MIKC^C^-type proteins, MIKC* -type proteins tend to have longer I domains and fewer conserved K domains. MIKC^C^-type MADS-box genes are the most representative class of *MADS-box* genes, which play important and diverse roles in plant growth and development [3,8]. MIKC^C^-type MADS-box genes can be divided into 12 subclasses according to their phylogenetic relationships in *Arabidopsis thaliana* [9]. Compared with the type II lineage group, the type I genes have a simpler gene structure and lacks the K domain. They are thought to share a common ancestor with type I genes from animals and fungi, but their functions are generally not well understood [3,10]. Type I MADS-box genes can be further subdivided into Mα, Mβ, Mγ and Mδ in plants [11,12].

MADS-box genes play an important role in plant development. The most important role is as a major component of the well-known ABCDE model, which performs its function in regulating floral organs. Different combinations of A, B, C, D and E functions of *MADS-box* genes determine different floral organ characteristics: (sepals (A + E), petals (A + B + E), stamens (B + C + E), carpels (C + E) and ovules (D + E)). Class A genes mainly control the development of calyx, corolla and floral organs. B-type genes control the development of corolla and stamens, and also affect the development of calyx in a few plants. Class C genes control the development of three-wheeled floral organs of stamens, pistils and ovules, while only two-wheeled structures of stamens and pistils are controlled in some plants. Class D genes control the development of ovules, and class E genes are involved in the formation of floral organs during each round of flower development, and form a tetramer model complex with class A, B, and C genes. In Arabidopsis, the corresponding functional genes are class A, APETALA 1 (AP 1); class B, PISTILATA (PI) and AP 3; class C, AGAMOUS (AG); class D, SEEDSTICK/AGAMOUS-LIKE 11(STK/AGL 11); and E, SEPALLATA (SEP 1, SEP 2, SEP 3 and SEP 4) [3,13,14,15].

In addition to its important role in determining floral organ traits, MADS-box genes have also been found to be involved in the regulation of flowering time and flower initiation, such as SOC1, FLC, AGL and SVP [16,17,18,19,20,21,22,23]; as well as in fruit formation (FUL) [24,25] and root development (AGL12 and AGL17) [26,27].

*MADS-box* genes have been demonstrated to participate in the nutrient growth processes and various stress responses in different plants such as Arabidopsis [28], rice [29], wheat [30,31], and cabbage [32]. Therefore, the MADS-box protein family is an important transcription factor family, influencing almost the entire process of plant growth and development. Model plant species of the MADS-box family have been extensively studied, including rice (*Oryza sativa*), cabbage (*Brassica rapa*), poplar (*Populus trichocarpa*), wheat (*Triticum aestivum* L.), banana (*Musa acuminata*), and others.

Cucumber (*Cucumis sativus* L.) is an economically and nutritionally important vegetable crop cultivated worldwide. It is loved by people because of its good taste, high nutritional value and economic value. Although *MADS-box* genes exist as a superfamily, little is known about MADS-box genes in cucumber. Therefore, it is of great significance to identify the MADS-box gene family of cucumber. In previous studies, 43 MADS-box genes were identified in the V1 genome of cucumber [33]. With the update of the Cucurbitaceae genome database, the cucumber genome version has been updated to the V3 version. Therefore, it is necessary to re-identify and modify the members of the MADS-box gene family in cucumber. This work is helpful in the context of a more comprehensive study of cucumber MADS-box gene family members and provides a basis for further functional analysis to elucidate their roles in development.

## 2. Results

### 2.1. Characterization of MADS-Box from Chinese Long 9930 (V3 Version)

**Figure 1:**
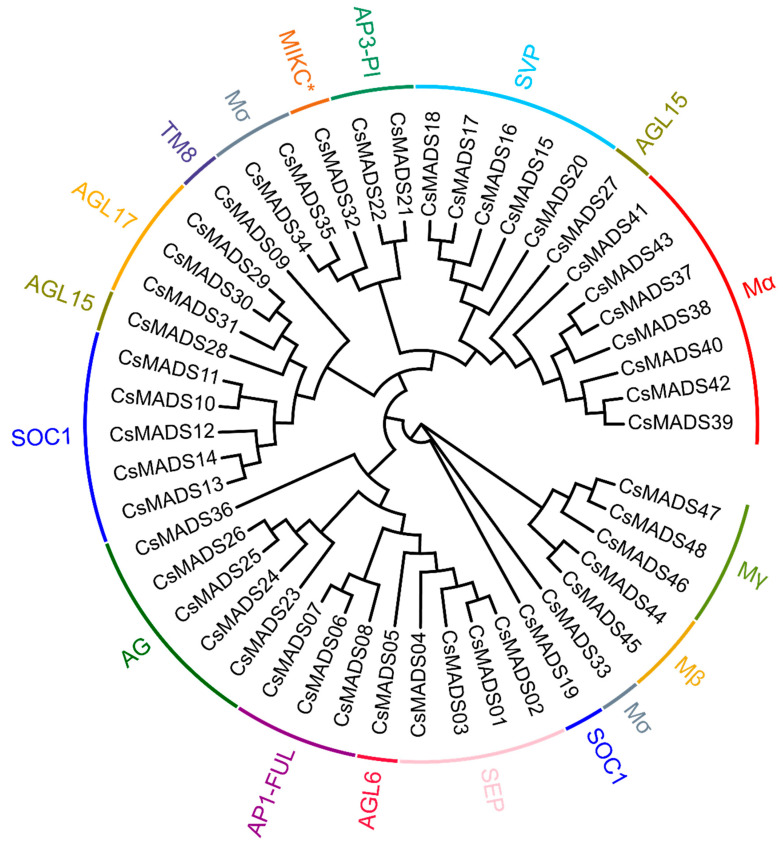
The phylogenetic tree of the MADS-box proteins from cucumber. These proteins were phylogenetically analyzed using MEGA7 software with 1000 bootstrap tests. Different colors represent different subgroups of the MADS-box family (AG (AGAMOUS) and SOC1 (SUPPRESSOR OF OVEREXPRESSION OF CO 1)) “*” represents a type of MICK subfamily used to emphasize specific changes and specific amino acid sequence variations.

In previous studies, we identified 43 *MADS-box* genes in the cucumber V1 genome [33]. With the update of the cucumber family genome database, the cucumber genome version has been updated to V3. Therefore, we re-identified and revised the *MADS-box* gene family members in cucumber. In the cucumber V3 version, we identified a total of 48 *MADS-box* genes, and found that *Csa014213* and *Csa025232* aligned to the gene *CsaV3_6G006010* in the cucumber V3 version database; similarly, *Csa014249* and *Csa026408* aligned to *CsaV3_1G009750*; while *Csa014140* and *Csa025231* aligned to *CsaV3_6G006020*. *Csa014213* and *Csa025232* have the same CDS sequence. *Csa014140* and *Csa025231* also have the same CDS sequence. The CDS sequence similarity between *Csa014140* and *Csa025231* is as high as 96%. These results indicate that there are some errors in the *MADS-box* genes identified in the V1 version. Therefore, there are actually 40 *MADS-box* genes in the V1 version, 48 in the cucumber V3 version, and the 48 *MADS-box* genes are divided into 14 subfamilies (Figure 1). The eight newly added genes are *CsaV3_6G052910*, *CsaV3_6G051220*, *CsaV3_5G040310*, *CsaV3_5G040370*, *CsaV3_6G051590*, *CsaV3_3G009400*, *CsaV3_3G016620* and *CsaV3_UNG063480* (Table 1).

### 2.2. Analysis of the Differences of Amino Acid Sequences of MADS-Box Family Members Between V1 and V3 Versions

Due to the difference in the number of members of the *MADS-box* gene family in the V1 and V3 versions, we analyzed the differences in the protein sequences of the 43 genes found in the V1 version compared to the V3 versions. The results show that there were no differences in the amino acid sequences of 14 genes in the two versions of the database. They were *Csa004117*, *Csa008448*, *Csa014140*, *Csa025231*, *Csa012879*, *Csa012099*, *Csa017355*, *Csa000939*, *Csa021069*, *Csa017317*, *Csa020265*, *Csa017909*, *Csa002566* and *Csa001552*. The amino acid sequences of 29 genes were significantly different between the two versions of the data (Table 2). See Dataset S1 for amino acid sequence information.

### 2.3. Analysis of Protein Motif Difference of MADS-Box

**Figure 2:**
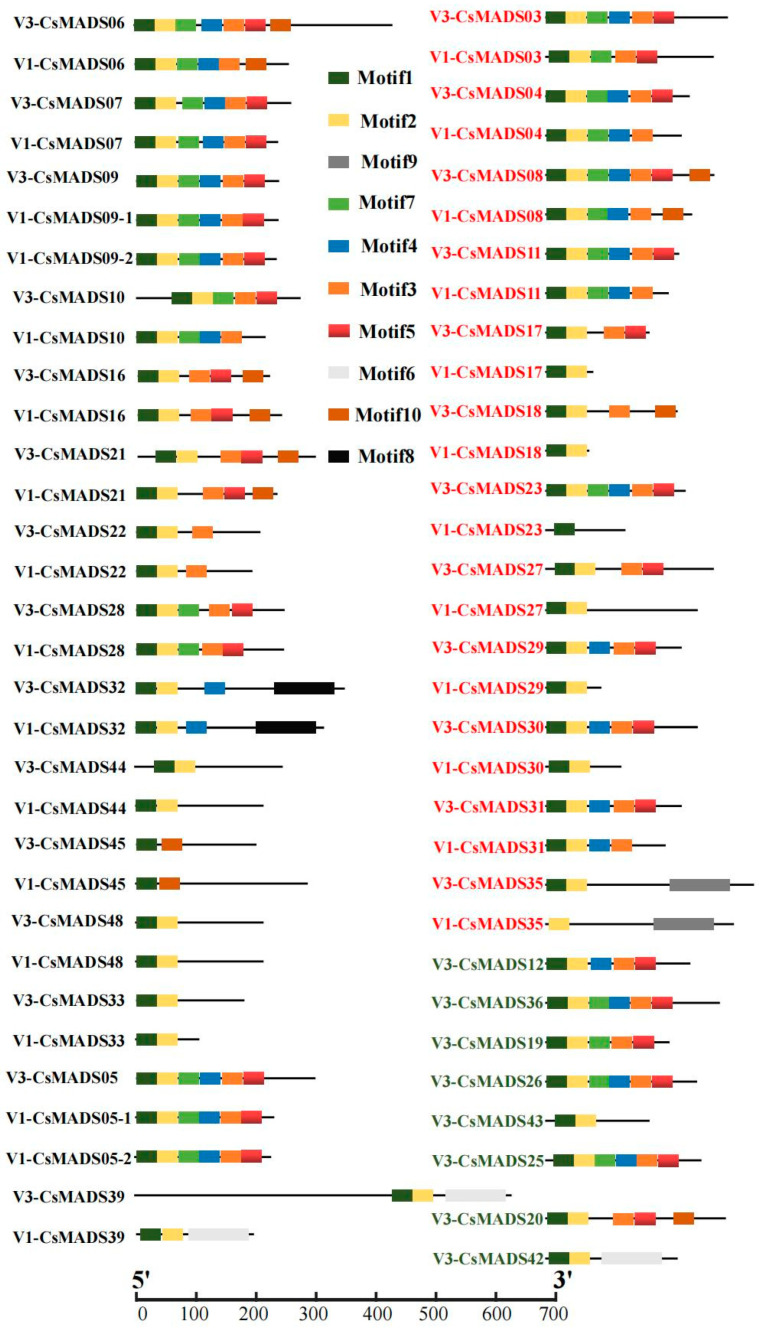
Analysis of differences in protein motifs of MADS-box in versions V1 and V3 (comparison of protein motifs with amino acid sequence differences between the two genomes).

In order to explore whether differences in amino acid sequence lead to the changes in protein conserved motifs, we compared and analyzed the protein conserved motifs of MADS-box family members in the V1 and V3 versions. The results show that the amino acid length of 15 family members changed, but their protein motifs did not change. They were CsMADS05, CsMADS06, CsMADS07, CsMADS09, CsMADS10, CsMADS16, CsMADS21, CsMADS22, CsMADS28, CsMADS32, CsMADS33, CsMADS39, CsMADS44, CsMADS45 and CsMADS48, respectively. The protein motifs of 12 family members changed, including CsMADS03, CsMADS04, CsMADS08, CsMADS11, CsMADS17, CsMADS18, CsMADS23, CsMADS27, CsMADS29, CsMADS30, CsMADS31 and, CsMADS35. Similarly, we analyzed the conserved motifs of the eight newly identified proteins, and found that CsMADS42 contains only two motifs (motif1, motif2), and CsMADS43 contains three motifs (motif1, motif2, motif6) (Figure 2). In addition, the conserved motifs of the remaining six proteins numbered five to six. Moreover, we found that motif1 and motif2 are present in most proteins, indicating that motif1 and motif2 are highly conserved (Figure 2). The amino acid sequence for each motif is presented in Figure S1.

### 2.4. CsMADS Multiple Sequence Alignment

**Figure 3:**
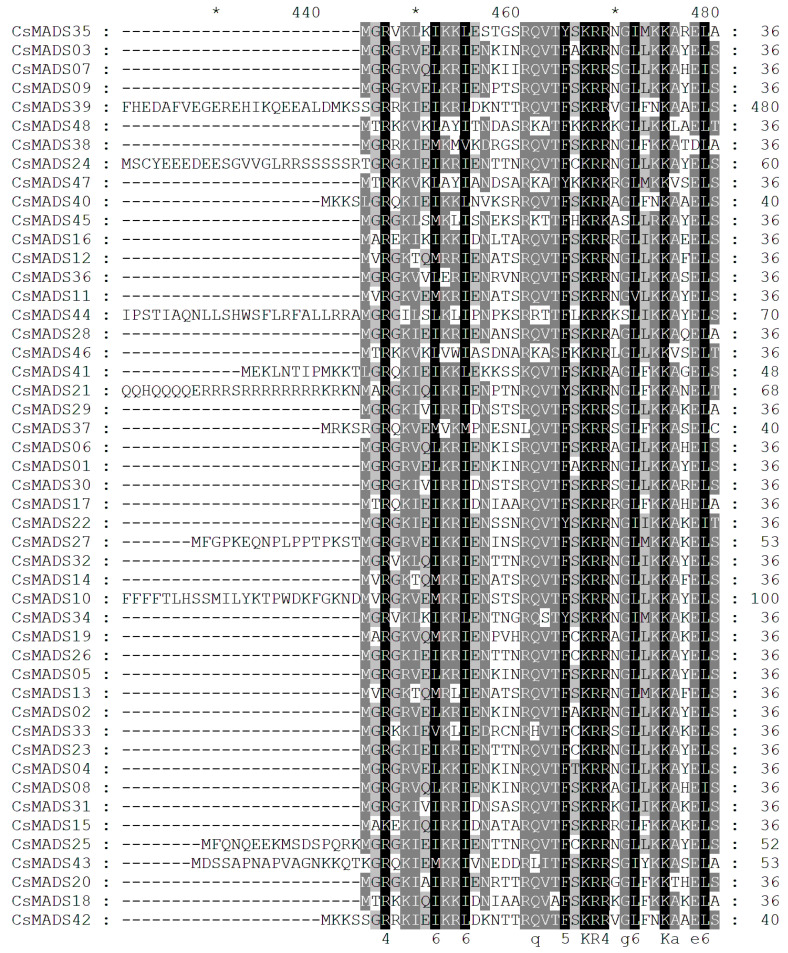
The multiple protein sequence alignment of the domains of MADS from cucumber.Conserved sequences are highlighted in black and grey shading; the black shading represents completely conserved sequences, while the grey shading represents incompletely conserved sequences. Black region: similarity over 80%. Grey region: similarity over 60–80%. “*” from left to right represents 430, 450, 470.

*MADS-box* genes have a highly conserved DNA binding domain, namely MADS box. By analyzing the amino acid sequence, we found that CsMADS contains a highly conserved MADS box (Figure 3).

### 2.5. Phylogenetic Relationship and Gene Structure Analysis of MADS-Box Genes

**Figure 4:**
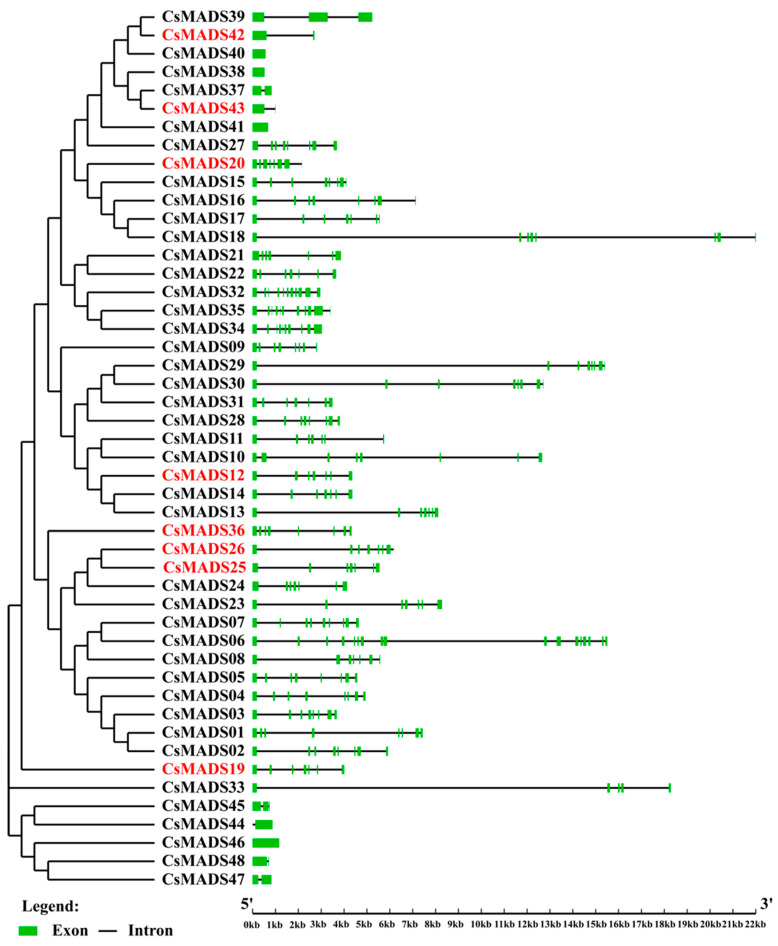
Phylogenetic tree and gene structure of MADS family members in *C. sativus*. The phylogenetic tree was constructed using the neighbor-joining (NJ) method with 1000 bootstrap replicates, based on the alignment of the identified MADS proteins in *C. sativus*. The gene structures of the 48 MADS genes identified in *C. sativus* were generated utilizing the Gene Structure Display Server v.2.0. In the structures, red represents the new genes in version of V3, the green box represents the exon, and the black line represents the intron.

The genome annotation information of the V1 cannot be obtained. Therefore, it is impossible to analyze the differences in the gene structures of the *MADS-box* family in the V1 and V3 versions, so only the structure of the *CsMADS* in the V3 version is analyzed. The results show that the numbers of introns in *CsMADS* varied greatly, ranging from 0 to 10. *CsMADS38*, *CsMADS40*, *CsMADS41*, *CsMADS44*, and *CsMADS46* have no introns, and these all belong to type I genes in the Mα and Mβ subfamilies. Type II genes, also known as MIKC genes, contain a large number of introns (CsMADS01-CsMADS32) and have similar structures (Figure 4).

### 2.6. Chromosome Distribution Analysis of CsMADS-Box Genes

**Figure 5:**
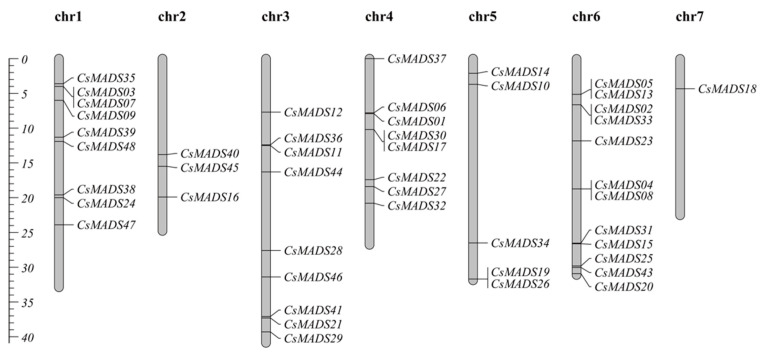
Chromosomal location of *CsMADS* genes.

The chromosome distribution analysis of the cucumber *CsMADS* gene shows that the distribution of the *CsMADS* gene on seven chromosomes was uneven (Figure 5). Specifically, there are nine *CsMADS* genes on chromosome 1 and chromosome 3, three *CsMADS* genes on chromosome 2, eight *CsMADS* genes on chromosome 4, and five *CsMADS* genes on chromosome 5. The number of *CsMADS* genes on chromosome 6 is the largest, with 12 *CsMADS* genes, and the number of *CsMADS* genes on chromosome 7 is the smallest, with only one *CsMADS* gene (Figure 5).

### 2.7. Analysis of Cis-Acting Elements in Gene Promoter Region

**Figure 6:**
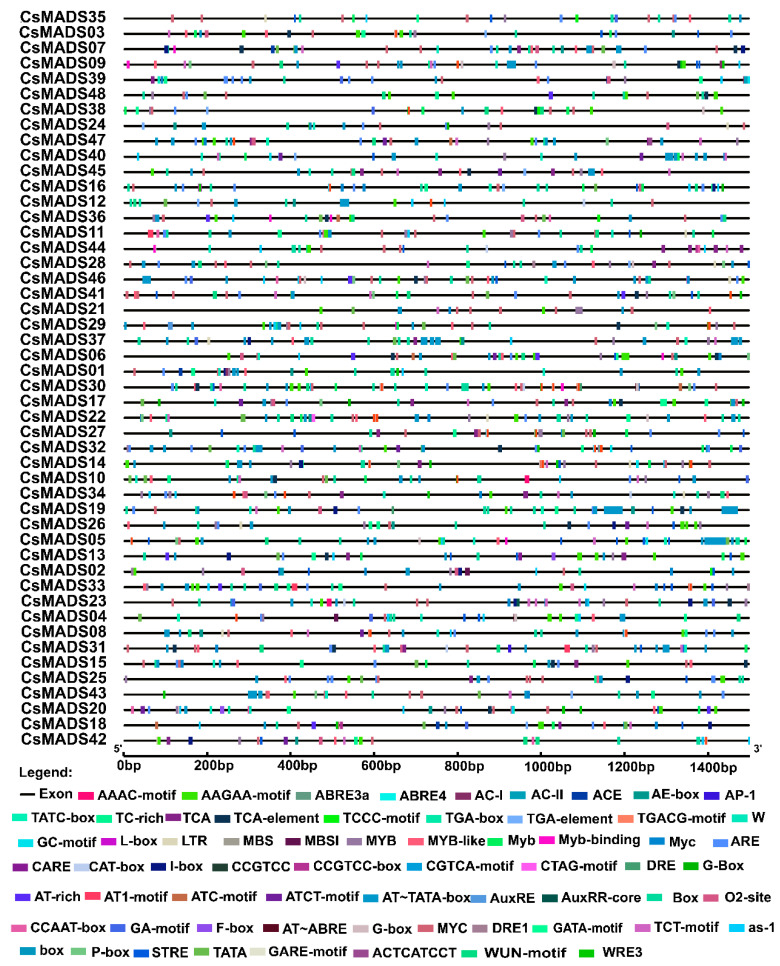
Predicted cis-elements in promoter regions of *CsMADS* genes. The promoter region was defined as a 1.5 kb sequence upstream of the translation initiation codon of the MADS gene. Identification of cis-acting elements using the online tool Plant CARE. Different types of cis-acting elements are represented by closed boxes of different colors.

The analysis of the 1500 bp upstream sequence of the *CsMADS* gene revealed a variety of cis-acting elements (Figure 6). For example, there were hormone response elements (ABRE, CGTCA-motif, GARE-motif, TCA-element, TATC~box, TCA-element, TGACG motif, etc.), stress corresponding components (MBS, MBSI, etc.), and other important response elements in plant growth and development (ARE, CAT-box, LTR, GC-motif, O2-site, RE-element, TC~rich). This shows that the MADS-box family is widely involved in the growth and development of plants (Figure 6).

### 2.8. Expression Patterns of CsMADS in Different Tissues

**Figure 7:**
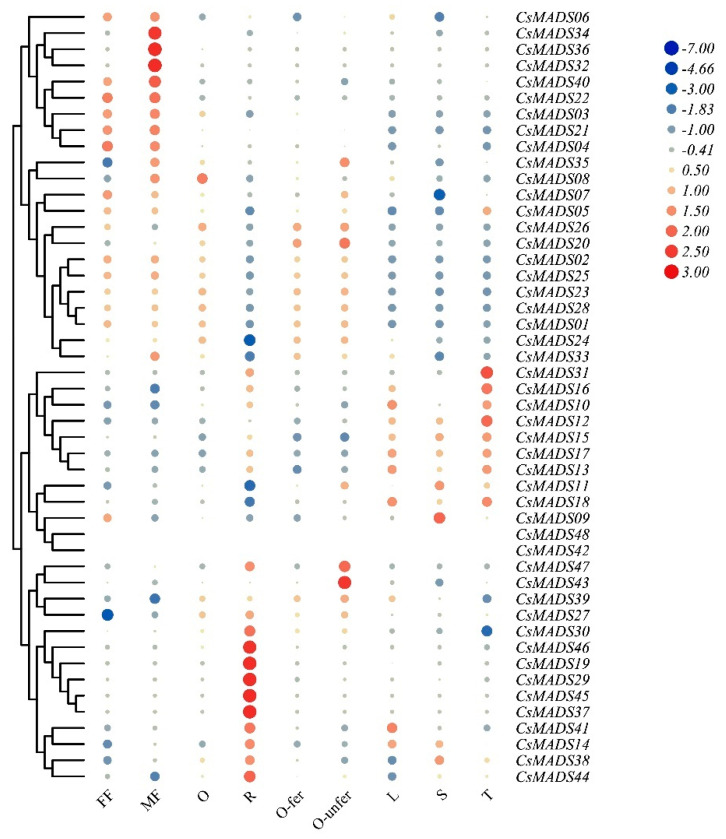
Temporal–spatial expression of cucumber *MADS* genes. Heatmap displaying the expression profiles of *CsMADS* genes in nine different cucumber tissues. The RNA-seq datasets with accession number PRJNA80169 were obtained from the Cucurbit Genomics Data website. The color scale represents Log_2_(FPKM) values, where blue and red indicate low and high expression levels, respectively. The FPKM values of *CsMADS* genes can be found in Table S1. R, root; S, stem; L, leaf; FF, female flower; MF, male flower; O, unexpanded ovary; O-fer, expanded fertilized ovary; O-unfer, expanded unfertilized ovary; T, tendril.

This part aims to study the role of *MADS* genes in cucumber development by analyzing public RNA-seq data of different tissues. The results show that, compared with other tissues, the expression of most *CsMADS* genes in cucumber stems was relatively low (Figure 7). In contrast, *CsMADS10*, *CsMADS12*, *CsMADS16* and *CsMADS31* were highly expressed in tendrils. *CsMADS03*, *CsMADS22*, *CsMADS32*, *CsMADS34*, *CsMADS36* and *CsMADS40* were highly expressed in female flowers, indicating their potential roles in flower differentiation and development. *CsMADS14*, *CsMADS19*, *CsMADS29*, *CsMADS30*, *CsMADS37*, *CsMADS38*, *CsMADS41*, *CsMADS44*, *CsMADS45* and *CsMADS46* were mainly expressed in roots, indicating that they may be involved in regulating ion transport in the underground part of cucumber (Figure 7).

### 2.9. Expression Profiles of CsMADS Genes Under Abiotic and Biotic Stresses

Although *MADS* genes in many species have been identified to be involved in a variety of stress responses, they have not been studied in cucumber. In this study, based on the public transcriptome information, the expression patterns of *CsMADS* genes under different stress conditions such as salt, heat, downy mildew (DM, *Pseudoperonospora cubensis*) and powdery mildew (PM, *Podosphaera fusca*) were analyzed to explore the role of *CsMADS* genes under different stress conditions.

**Figure 8:**
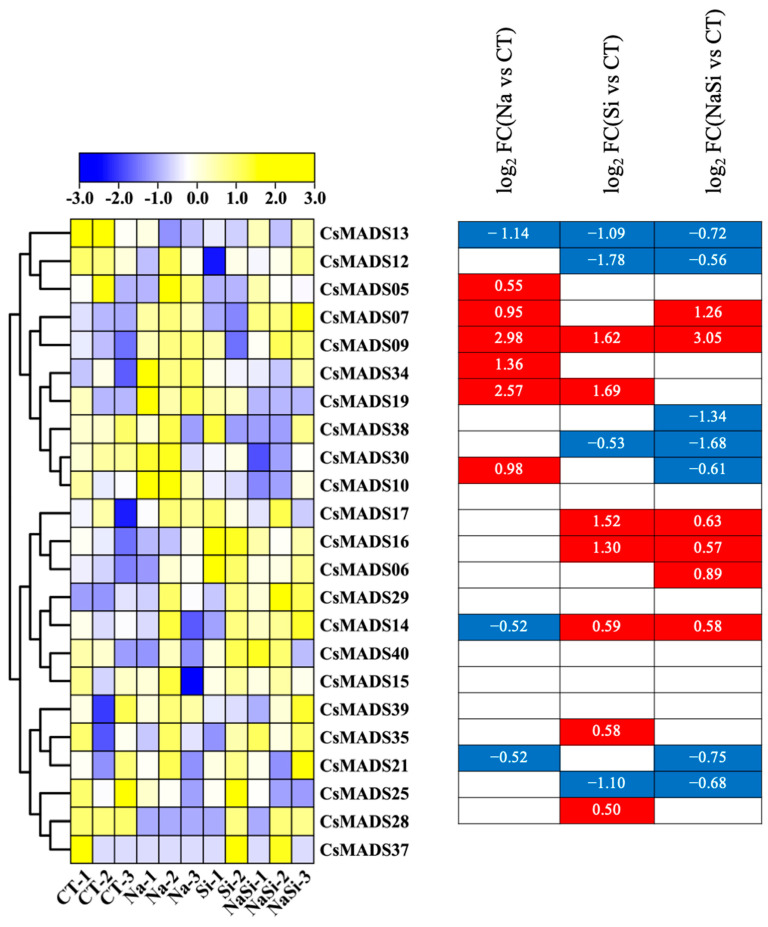
Expression profiles of *CsMADS* genes in response to salt stress treatments: A range of −3.00 to 3.00 was artificially set with the color scale limits according to the normalized values. The color scale shows increasing expression levels from blue to yeollow. Blue region: Down-regulated Red region: Up-regulated. The FPKM value of *CsMADS* genes under salt are listed in Table S2.

Firstly, the role of the *CsMADS* gene under salt stress was analyzed. The results are presented in the form of heat maps (Figure 8). Under NaCl treatment, most genes were up-regulated; only a small number of genes were down-regulated, including *CsMADS13*, *CsMADS25* and *CsMADS40*. Silicon (Si) is considered to be an essential element for plant growth and development, and plays a significant role in promoting plant growth and development and enhancing stress resistance. We observed that gene expression undergoes significant changes exclusively under the Si treatment condition. The expressions of *CsMADS06*, *CsMADS09*, *CsMADS16*, *CsMADS19*, *CsMADS21*, *CsMADS37* and *CsMADS40* were up-regulated (Figure 8). In contrast, the expression of *CsMADS12*, *CsMADS13*, *CsMADS28* and *CsMADS30* were down-regulated. It is worth noting that the expressions of *CsMADS06*, *CsMADS07*, *CsMADS09*, *CsMADS16*, *CsMADS29* and *CsMADS40* were up-regulated under the treatment of NaCl and silicon(Si), suggesting that they play an important role in the process of plant resistance to salt stress (Figure 8).

**Figure 9:**
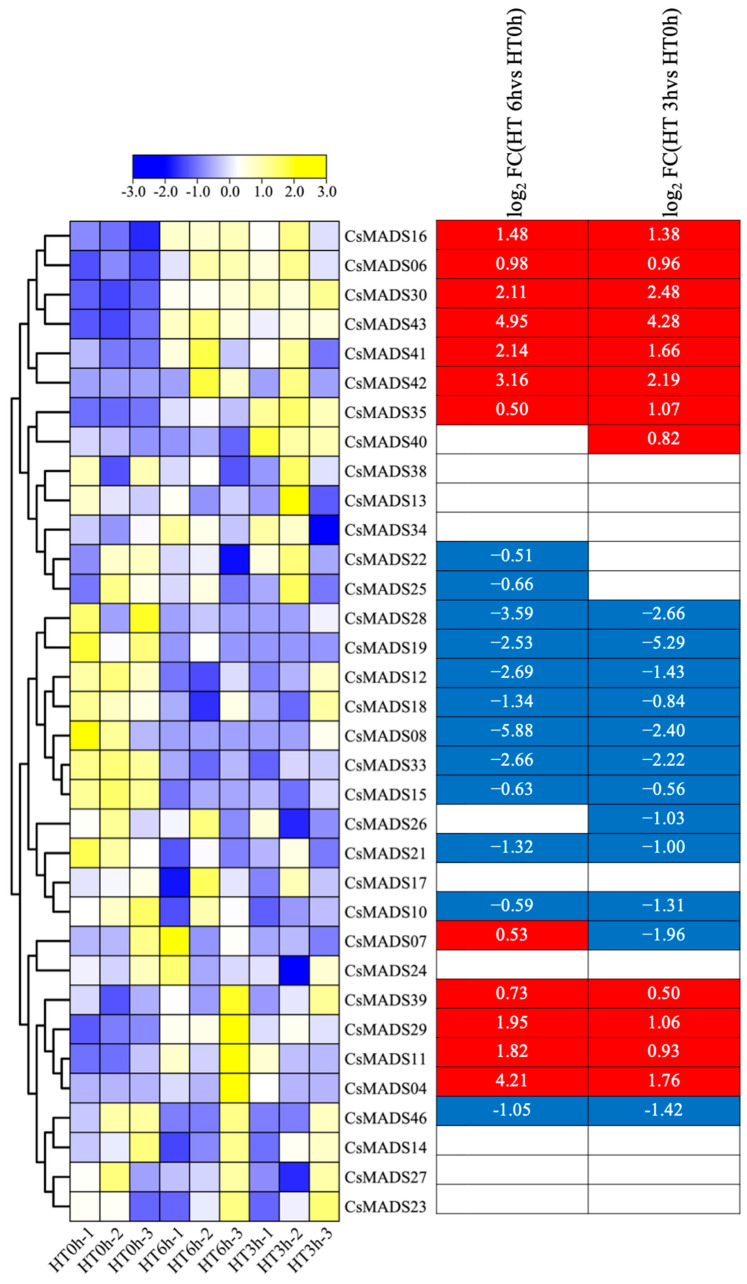
Expression profiles of *CsMADS* genes in response to heat stress treatments: A range of −3.00 to 3.00 was artificially set with the color scale limits according to the normalized values. The color scale shows increasing expression levels from blue to yeollow. Blue region: Down-regulated Red region: Up-regulated.The FPKM value of *CsMADS* genes under heat stress are listed in Table S3.

We also analyzed the responses of *CsMADS* genes to heat stress. The expressions of *CsMADS04*, *CsMADS06*, *CsMADS16*, *CsMADS29*, *CsMADS30*, *CsMADS35*, *CsMADS39, CsMADS41*, *CsMADS42* and *CsMADS43* were significantly up-regulated in both three-hour and six-hour heat and high temperature treatments. The expressions of *CsMADS08, CsMADS10*, *CsMADS12*, *CsMADS15*, *CsMADS18*, *CsMADS19*, *CsMADS21*, *CsMADS28*, *CsMADS33* and *CsMADS46* were significantly down-regulated. In addition, the results show that the expression of CsMADS07 was significantly down-regulated after 3 h of high-temperature treatment, but significantly up-regulated after 6 h of high-temperature treatment (Figure 9).

**Figure 10:**
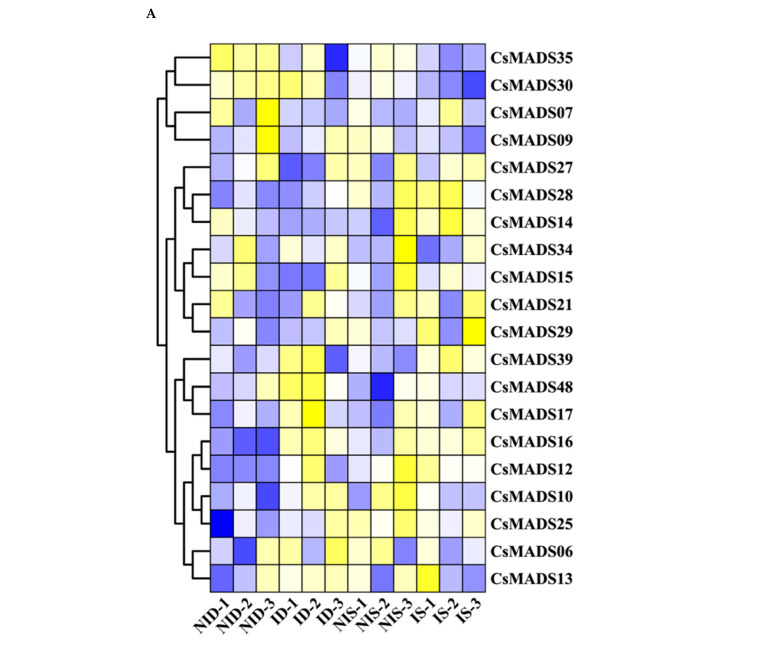

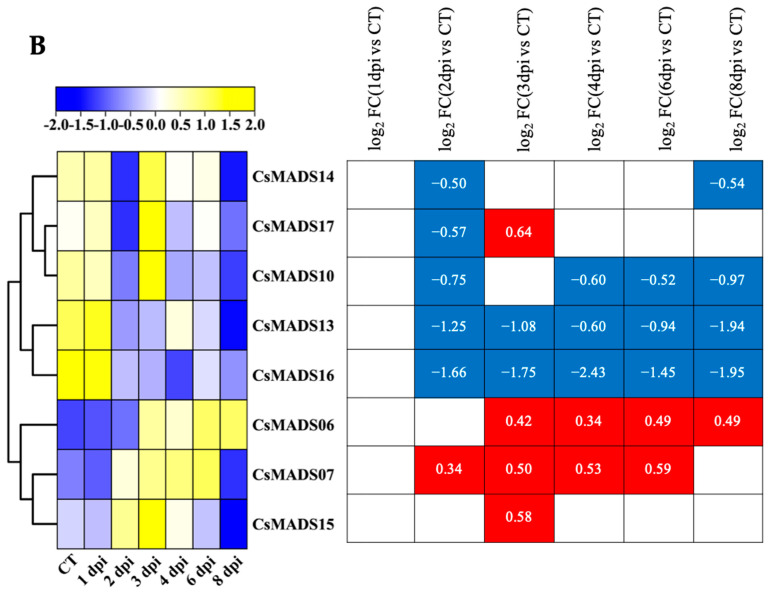
Expression analysis of Cs*MADS* under biotic stresses: The transcriptional levels of Cs*MADS* genes after infection with powdery mildew (PM) for 48 h (**A**) and with downy mildew (DM) for 1–8 days post-inoculation (**B**) are shown on the heatmaps. A range of −3.00 to 3.00 was artificially set with the color scale limits according to the normalized values. The color scale shows increasing expression levels from blue to red. ID, PM-inoculated susceptible cucumber line D8 leaves; NID, non-inoculated D8 leaves; IS, PM-inoculated resistant cucumber line SSL508–28 leaves; NIS, non-inoculated SSL508–28 leaves; CT, without inoculation; DPI, days post inoculation; FC, fold-change. Blue region: Down-regulated Red region: Up-regulated. The FPKM value of *CsMADS* genes under powdery mildew (PM) and downy mildew (DM) are listed in Table S4.

In order to explore the role of *CsMADS* in biological stress resistance, we used the RNA-Seq database to analyze the expression of *CsMADS*. The results show that *CsMADS17*, *CsMADS39* and *CsMADS48* were significantly up-regulated in susceptible and resistant cucumber lines after inoculation with powdery mildew (PM). On the contrary, the expression of *CsMADS09* was down-regulated (Figure 10A). In addition, we found that the expression of *CsMADS25* was significantly up-regulated in susceptible cucumber lines, indicating that the gene may play an important role in the mechanism of cucumber resistance to powdery mildew.

In the transcriptome data of cucumber seedlings inoculated with DM, only eight *MADS* genes were detected. *CsMADS06* and *CsMADS07* were up-regulated at most treatment time points, while *CsMADS10*, *CsMADS13* and *CsMADS16* were down-regulated at most treatment time points (Figure 10B).

## 3. Discussion

The *MADS-box* gene is an important transcriptional regulator in eukaryotes, which is involved in the regulation of growth and development and signal transduction processes. It has been widely identified in many species [32,33]. Although the *MADS-box* gene of cucumber has been previously identified, its comprehensive identification and characterization are limited due to the low quality of the genome. In addition, the function of MADS-box family in signal transduction and response to different stress conditions is also relatively insufficient. With the update of the Cucurbitaceae genome database, the cucumber genome has been upgraded. Therefore, it is necessary to re-identify and modify the members of the *MADS-box* gene family in cucumber to further explore its variation and potential functions, and to elucidate its role in cucumber development.

In previous studies, 43 *CsMADS-box* genes were identified in the cucumber V1 database [33], while 48 *CsMADS-box* genes were identified in the cucumber V3 database. By analyzing the 43 *CsMADS-box* genes in the V1 version, we found that *Csa014213* and *Csa025232* had the same CDS sequence, as well as *Csa014249* and *Csa026408*. In addition, the CDS sequence similarity of *Csa014140* and *Csa025231* was as high as 96%, indicating that they may actually be the same gene (Table 1). These results indicate that there are some errors in previous studies, so it is necessary to re-identify and correct the members of the *MADS-box* gene family in cucumber.

By analyzing the differences in protein sequences and motifs, we found that more than half of the amino acid sequences were significantly different between the two versions of the data (Table 2). Specifically, there were no differences in the amino acid sequences of 14 genes, which were *Csa004117*, *Csa008448*, *Csa014140*, *Csa025231*, *Csa012879*, *Csa012099*, *Csa017355*, *Csa000939*, *Csa021069*, *Csa017317*, *Csa020265*, *Csa017909*, *Csa002566* and *Csa001552*. In addition, the amino acid length of 15 family members changed, but their protein motifs did not change (CsMADS05, 06, 07, 09, 10, 16, 21, 22, 28, 32, 33, 39, 44, 45 and 48). Besides this, we found that motif1 and motif2 are highly conserved in most *CsMADS* genes (Figure 2). Type I (Mα, Mβ and Mγ) MADS-box genes usually lack introns or have only one intron, and their gene structure is very simple (Figure 4). In contrast, the gene structure of type II genes (MIKC and Mδ) seems to be more complex, including multiple exons and introns. Studies have shown that genes containing multiple introns are usually more conserved [34], so type I genes may not be as conserved as type II MADS-box genes. In addition, studies have shown that a small number of introns help genes respond quickly to various stresses and activate down-regulated genes [35]. The presence of introns may lead to alternative splicing, thereby delaying the response to stress, and type I genes may respond to stress earlier. Chromosomal localization analysis showed that *CsMADS-box* genes were unevenly distributed on seven chromosomes of cucumber (Figure 5).

We also identified 10 important cis-acting elements of the MADS-box family, most of which are related to plant hormones. Hormone response elements (ABRE, CGTCA-motif, GARE-motif, TCA-element, TATC-box, TCA-element, TGACG motif) were highly enriched in the promoter region of *CsMADS* gene, indicating that the *CsMADS* gene may be involved in the regulation of various hormone responses (Figure 6). Meristem response elements (ARE, CAT-box, LTR, GC-motif, O2-site, RE-element, TC-rich) are mainly identified in type II genes, suggesting that type II *MADS-box* genes have a function in determining meristem and floral organ identity in cucumber, which is in accordance with *Callicarpa americana* [36].

Understanding gene expression is essential to reveal the molecular mechanism of biological development [37]. *MADS-box* genes are widely thought to be associated with floral organ development and identity determination in plants. Previous studies have shown that AOAMOUS (AG) and APETALA1 (AP1) gene family members are mainly expressed in flowers, fruits and buds in species such as tomato [38], cotton [39], watermelon [40] and soybean [41]. The results of this study are consistent with previous observations. AG gene family members (*CsMADS23*, *24*, *25*, *26* and *36*) and AP1 gene family members (*CsMADS06*, *07* and *08*) are mainly expressed in cucumber flowers. Moreover, we found that 10 other *MADS* genes (*CsMADS14*, *19*, *29*, *30*, *37*, *38*, *41*, *44*, *45* and *46*) were highly expressed in roots, indicating that they may play an important role in plant growth and ion transport (Figure 7).

In addition to regulating the characteristics of floral organs and their meristems in plant flower development, *MADS-box* genes have also been found to be involved in a variety of stress responses [42]. For example, *DgMADS114* and *DgMADS115* can enhance the resistance of transgenic Arabidopsis to PEG, NaCl, ABA and high-temperature stress [43]. Under ABA treatment, the expression levels of *MsMADS001* and *MsMADS075* gradually increased with time, while *MsMADS075* showed a trend of increasing first and then decreasing under drought treatment, which is consistent with the results of RNA-Seq and qRT-PCR analysis, indicating that they may play an important role in stress response [44]. In addition, the expression level of *TaMADS19* was significantly increased after inoculation with wheat stripe rust. After inoculation with powdery mildew, the expression of *TaMADS117* was significantly reduced. The expression levels of *TaMADS121*, *93* and *21* were significantly increased under phosphorus deficiency stress. Under high temperature stress, the expression levels of *TaMADS63* and *41* were significantly reduced [45]. In this study, we found that the expression levels of 26,18,8 and 10 *CsMADS-box* genes showed significant change after high temperature, NaCl, silicon, downy mildew and powdery mildew treatments (Figure 8, Figure 9 and Figure 10). Only two genes, *CsMADS07* and *CsMADS16*, showed responses to all tested stress conditions, suggesting their pivotal role in conferring cucumber’s resistance to multiple environmental stresses. These findings imply that *CsMADS07* and *CsMADS16* might function as key regulatory factors in the plant’s stress tolerance mechanisms, possibly by modulating pathways involved in stress signal transduction, reactive oxygen species (ROS) regulation, or hormonal responses. Given their broad response to various abiotic stresses, these genes could be valuable targets for genetic improvement programs aimed at enhancing cucumber’s resilience to climate change-induced stressors. In summary, this study highlights the significant contribution of *CsMADS* genes to cucumber growth and development, while providing new genetic resources for future breeding efforts focused on stress resistance in cucumber.

## 4. Material and Methods

### 4.1. Identification of MADS-Box Genes in Cucumber

The cucumber genome data were downloaded from the Cucurbit Genomics Database (http://cucurbitgenomics.org/) (accessed on 1 March 2024) and NCBI (https://www.ncbi.nlm.nih.gov/). The hidden Markov model (HMM) profile files of the MADS-box conserved domain (PF00319) were downloaded from the Pfam database (http://pfam.xfam.org/). The MADS-box genes of cucumber were identified from the genome database using HMMER 3.0 with the default parameters and a cutoff value of 0.01. All CsMADS-boxs were further examined to confirm the MADS-box conserved domain through the CDD, Pfam, and SMART online tools. All *CsMADS-box* genes were named according to their locations on seven cucumber chromosomes.

### 4.2. Gene Structure and Motif Analysis

The CDS sequences and genomic data for *CsMADS-box* genes retrieved from the C. sativus genome database (http://cucurbitgenomics.org/organism/20) (accessed on 8 April 2025) were visualized using the Gene Structure Display Server online tool (http://gsds.cbi.pku.edu.cn/) [46]. The conserved motifs of CsMADS-box proteins were then identified with MEME 4.9.1 (http://meme-suite.org/) [47] and visualized with WebLogo (http://weblogo.berkeley.edu/logo.cgi) (accessed on 8 April 2025) [48]. The total number of motifs (nmotifs) is 10, the minimum length of motifs (minw) is 6 amino acids, and the maximum length of motifs (maxw) is 10 amino acids.

### 4.3. Phylogenetic Analysis and Multiple Sequence Alignment

The protein sequences of MADS-box in cucumber were uploaded to the MEGA software (v7.0) to be aligned using ClustalW 2.x, and the phylogenetic relationships among all MADS-box proteins were examined via the neighbor-joining method with 1000 bootstrap replicates. Then, the phylogenetic trees were landscaped in Evolview (https://evolgenius.info//evolview-v2/#login, accessed on 8 April 2025).

### 4.4. Gene Duplication Analysis and Genome Distribution

*CsMADS-box* loci were extracted from the cucumber genome database (http://cucurbitgenomics.org/organism/20) and their locations on chromosomes were visualized using MapChart 3.x [49].

### 4.5. Analysis of Promoter Regions of CsMADS-Box Genes

The 1.5-kb sequences upstream of the initiation codons (ATG) of *CsMADS-box* genes were obtained from the cucurbit genomics data website (http://cucurbitgenomics.org/organism/20), and analyzed for cis-elements in the promoter region using the online tool PlantCARE [50].

### 4.6. Transcriptome Analysis of CsMADS-Box Genes in Cucumber

The expression patterns of the *CsMADS* genes were analyzed using the transcriptomic data of the roots, stems, leaves, flowers, ovaries, and tendrils of cucumber. The published RNA-Seq data (SRA046916) [51] were downloaded from the Cucurbit Genomics Database (http://cucurbitgenomics.org/). The remapped clean tags and the recalculated FPKM values were cited to analyze the expression patterns of the *CsMADS*. The genome-wide expression of the *CsMADS* genes was shown on a heatmap using TBtools v2.x [52]. The heatmap values were calculated according to the following steps: the fold change values of the FPKM value of the treatment group and the control group were calculated first, and then the logarithm based on two of the fold change values was taken.

### 4.7. Transcriptome Analysis of CsMADS in Response to Abiotic and Biotic Stresses

The publicly available transcriptomic data of cucumber treated with salt (GSE116265) [53], heat (GSE151055) [54], DM (SRP009350) [55], and PM (GSE81234) [56] were downloaded from NCBI (https://www.ncbi.nlm.nih.gov/) to analyze the expression patterns of *CsMADS* under different stresses. After aligning the gene IDs to the cucumber genome, the genome-wide expression of the *CsMADS* genes was shown on a heatmap using TBtools V2.056. For the transcriptome analysis of the *CsMADS*, a threshold of FDR (or *p*-value) ≤ 0.05 and an absolute value of log2 (fold-change) ≥ 1 or log2 (fold-change) ≤ −1 were used to define DEGs.

### 4.8. The Methodology Was Involved in the Development of the V1 and V3 Versions

Development Methodology of V1 Version: The V1 version of the cucumber genome was primarily developed using early sequencing technologies, including Sanger sequencing and early Illumina short-read sequencing. The assembly process in this version relied on lower coverage and fewer assembly strategies, which resulted in some issues such as incomplete gene lengths, inaccurate gene spacing, and assembly problems in repetitive regions. Although the V1 version provided an initial genomic framework, it had limitations in gene annotation and sequence completeness, particularly for longer genes and complex genomic regions.

Development Methodology of V3 Version: In contrast, the V3 version of the cucumber genome was developed using a more advanced combination of Illumina short-read sequencing and PacBio long-read sequencing technologies. These advancements provided higher coverage and longer read lengths, allowing for better handling of repetitive regions and structural variations within the genome. The V3 version also utilized more sophisticated gene prediction and annotation tools, incorporating extensive transcriptomic data and experimental validation to significantly improve the accuracy of gene annotations.

## 5. Conclusions

In this study, we re-identified and revised the members of the *MADS-box* gene family. In the updated version (V3), 48 *CsMADS-box* genes were identified—8 more than in the V1 version. In different versions, most amino acid sequences are significantly different. The further analysis of conserved motifs revealed that the conserved motifs of 12 genes were changed. In addition, we found that motif1 and motif2 are highly conserved. Gene structure analysis showed that most of the type I genes did not contain introns. The gene structure of type II is conserved, with a notably high number of introns. The *CsMADS-box* gene is distributed across seven chromosomes in cucumber. The promoter region of the *CsMADS-box* gene contains response elements and other elements related to plant growth and development. The expression pattern analysis of *CsMADS-box* in different tissues has shown that they were likely to be involved in the growth and morphogenesis of cucumber. Furthermore, transcriptome data under different stress conditions reveal the expression of *CsMADS-box* under abiotic and biotic stresses, and show that *CsMADS07* and *CsMADS16* responded to all four stresses. This study provides data and a theoretical reference for the potential role of *CsMADS* in cucumber stress resistance breeding.

## Figures and Tables

**Table 1:** Comparison of the number of MADS-box families in the Cucumber V1 and V3 versions.

Serial No.	Gene Name	Gene ID(V3)	Gene ID(V1)	Chromosamal Location	Group	Subfamily
1	*CsMADS01*	CsaV3_4G010090.1	Csa004117	4	MIKC	SEP
2	*CsMADS02*	CsaV3_6G008200.1	Csa008448	6	MIKC	SEP
3	*CsMADS03*	CsaV3_1G006210.1	Csa004591	1	MIKC	SEP
4	*CsMADS04*	CsaV3_6G033790.1	Csa013129	6	MIKC	SEP
5	*CsMADS05*	CsaV3_6G006010.1	Csa014213, Csa025232	6	MIKC	AGL6
6	*CsMADS06*	CsaV3_4G010080.1	Csa004120	4	MIKC	AP1-FUL
7	*CsMADS07*	CsaV3_1G006220.1	Csa004592	1	MIKC	AP1-FUL
8	*CsMADS08*	CsaV3_6G033800.1	Csa013130	6	MIKC	AP1-FUL
9	*CsMADS09*	CsaV3_1G009750.1	Csa014249, Csa026408	1	MIKC	TM8
10	*CsMADS10*	CsaV3_5G005600.1	Csa012493	5	MIKC	SOC1
11	*CsMADS11*	CsaV3_3G016650.1	Csa021114	3	MIKC	SOC1
12	*CsMADS12*	CsaV3_3G009400.1		3	MIKC	SOC1
13	*CsMADS13*	CsaV3_6G006020.1	Csa014140, Csa025231	6	MIKC	SOC1
14	*CsMADS14*	CsaV3_5G003360.1	Csa012879	5	MIKC	SOC1
15	*CsMADS15*	CsaV3_6G045010.1	Csa012099	6	MIKC	SVP
16	*CsMADS16*	CsaV3_2G030300.1	Csa003859	2	MIKC	SVP
17	*CsMADS17*	CsaV3_4G014770.1	Csa017496	4	MIKC	SVP
18	*CsMADS18*	CsaV3_7G006940.1	Csa003446	7	MIKC	SVP
19	*CsMADS19*	CsaV3_5G040310.1		5	MIKC	SVP
20	*CsMADS20*	CsaV3_6G052910.1		6	MIKC	SVP
21	*CsMADS21*	CsaV3_3G045590.1	Csa017887	3	MIKC	AP3-PI
22	*CsMADS22*	CsaV3_4G028010.1	Csa011135	4	MIKC	AP3-PI
23	*CsMADS23*	CsaV3_6G015770.1	Csa000681	6	MIKC	AG
24	*CsMADS24*	CsaV3_1G032920.1	Csa017355	1	MIKC	AG
25	*CsMADS25*	CsaV3_6G051220.1		6	MIKC	AG
26	*CsMADS26*	CsaV3_5G040370.1		5	MIKC	AG
27	*CsMADS27*	CsaV3_4G028880.1	Csa021473	4	MIKC	AGL15
28	*CsMADS28*	CsaV3_3G031900.1	Csa020302	3	MIKC	AGL15
29	*CsMADS29*	CsaV3_3G048150.1	Csa002117	3	MIKC	AGL17
30	*CsMADS30*	CsaV3_4G014700.1	Csa017500	4	MIKC	AGL17
31	*CsMADS31*	CsaV3_6G044810.1	Csa012111	6	MIKC	AGL17
32	*CsMADS32*	CsaV3_4G030750.1	Csa015983	4	MIKC	MIKC*
33	*CsMADS33*	CsaV3_6G008210.1	Csa008449	6	Mσ	
34	*CsMADS34*	CsaV3_5G032860.1	Csa000939	5	Mσ	
35	*CsMADS35*	CsaV3_1G005580.1	Csa004560	1	Mσ	
36	*CsMADS36*	CsaV3_3G016620.1		3	AG	
37	*CsMADS37*	CsaV3_4G000010.1	Csa021069	4	Mα	
38	*CsMADS38*	CsaV3_1G032570.1	Csa017317	1	Mα	
39	*CsMADS39*	CsaV3_1G015790.1	Csa007119	1	Mα	
40	*CsMADS40*	CsaV3_2G016620.1	Csa020265	2	Mα	
41	*CsMADS41*	CsaV3_3G045410.1	Csa017909	3	Mα	
42	*CsMADS42*	CsaV3_UNG063480.1			Mα	
43	*CsMADS43*	CsaV3_6G051590.1		6	Mα	
*44*	*CsMADS44*	*CsaV3_3G020270.1*	*Csa014962*	*3*	*Mβ*	
*45*	*CsMADS45*	*CsaV3_2G019470.1*	*Csa017249*	*2*	*Mβ*	
*46*	*CsMADS46*	*CsaV3_3G038110.1*	*Csa002566*	*3*	*Mγ*	
*47*	*CsMADS47*	*CsaV3_1G038060.1*	*Csa001552*	*1*	*Mγ*	
*48*	*CsMADS48*	*CsaV3_1G017300.1*	*Csa007130*	*1*	*Mγ*	

**Table 2:** Comparison of the amino acid number of MADS-box in the cucumber V1 and V3 versions.

Gene Name	V1	V3	Amino Acid Sequence Similarity	Gene Name	V1	V3	Amino Acid Sequence Similarity
*Csa004117*	245	245	100%	*Csa011135*	191	211	
*Csa008448*	241	241	100%	*Csa017355*	254	254	100%
*Csa004591*	250	255		*Csa021473*	255	286	
*Csa013129*	227	241		*Csa020302*	204	250	
*Csa014213*	171	246		*Csa002117*	91	225	
*Csa025232*	171	246		*Csa017500*	122	235	
*Csa004120*	197	555		*Csa012111*	204	228	
*Csa004592*	248	261		*Csa015983*	323	350	
*Csa013130*	201	223		*Csa008449*	107	181	
*Csa014249*	203	202		*Csa000939*	339	339	100%
*Csa026408*	210	203		*Csa004560*	313	355	
*Csa012493*	182	282		*Csa021069*	228	228	100%
*Csa021114*	177	183		*Csa017317*	173	173	100%
*Csa014140*	221	221	100%	*Csa007119*	202	602	
*Csa025231*	221	221	100%	*Csa020265*	187	187	100%
*Csa012879*	222	222	100%	*Csa017909*	225	225	100%
*Csa012099*	228	228	100%	*Csa014962*	216	250	
*Csa003859*	245	230		*Csa017249*	294	202	
*Csa017496*	67	173		*Csa002566*	387	387	100%
*Csa003446*	67	221		*Csa001552*	225	225	100%
*Csa017887*	244	276		*Csa007130*	219	213	
*Csa000681*	135	373					

## Data Availability

The data presented in this study are available in this article and Supplementary Materials.

## Supplementary Materials

The following supporting information can be downloaded at: https://www.mdpi.com/article/10.3390/ijms26083800/s1.
