# Supplementary material for: Genome-Wide Reidentification and Expression Analysis of MADS-Box Gene Family in Cucumber
Source: Int J Mol Sci. 2025 Apr 17;26(8):3800. doi: 10.3390/ijms26083800 (PMC12027882; doi:10.3390/ijms26083800)
Supplement: Supplementary file 1 [file ijms-26-03800-s001.zip › Dataset S1.pdf]

>Csa004117
MGRGRVELKRIENKINRQVTFAKRRNGLLKKAYELSVLCDAEVALIIFSNRGKLYEFCSTSNMLKTLERYQKCSYG
AVEVTKPAKELESSYREYLKLKSRFESLQRTQRNLLGEDLGPLNSKELEQLERQLVSSLKQVRSTKTQYMLDQLSDL
QNKEQMLIETNRALQIKLEEISSRNNIRLTWDGGDQSMSYGPQNAQTQGFFQPLECNPTLQIGYTSAVSDQITS
TTTPTHAQQVNGFLPGWML
>Csa008448
MGRGRVELKRIENKINRQVTFAKRRNGLLKKAYELSVLCDAEVALIIFSNRGKLYEFCSSSSMLKTLERYQKCNYG
APEPNVSTREALELSSQQEYLKLKARYEALQRSQRNLLGEDLGPLSSKELESLERQLDMSLKQIRSTRTQYMLDQL
TDLQRKEHLLNEANKTLKQRLVEGYQVNALQLNQSADDMMYGRQQAQPPGDAFFHPLDCEPTLQIGYQPDPI
TVVTAGPSMNNFLPGWLP
>Csa004591
MGRGRVELKRIENKINRQVTFAKRRNGLLKKAYELSVLCDAELALIIFSNRGKLFEFCSGSSMTKTLEKYRRCSYGI
PNATHQPQSFDDYLNLKATVEFMQQSQRNLLGEDLGPLNAKELEQLEHQLETSLERIRSTKTQSLLEQLTELQRK
EQMLVEDNRGLKKKLEESSAQVAVAAAGAWGWEDGAGGHNMEYPSRGVASQSDAFFHPIVQPTPTLQIGYS
SIGSMGMNHIGSPSQNANNNAFHLGWMI
>Csa013129
MGRGRVELKKIENKINRQVTFTKRRNGLLKKAYELSILCDAEVALIIFSARGKLYEFSSSPSIAKTLERYERHSYGALE
ASLPPKDTERWYQEYLKLKAEVEALQYSQRRFLGEELDDLETKELDQLEIQLEMSLKQIRSTKEDKLLETNQALRK
KLEESSAAIHHTSWDSSEPNNLQYCRQPEAFLQLNNNIIALENSYNPTEVTNEENVVNSGADGNGLSSHWMLL
>Csa014213
MGRGRVELKRIENKINRQVTFSKRRNGLLKKAYELSVLCDAEVALIIFSTRGKLYEFGSAGTSKTLERYQRCCFSPQ
HNFAERETQNWFQEISKLKAKYESLCRTHRHLLGEDLGPLSVKELQNLEKQLEAALSQARQRKTQIMIEQMENL
RKKERQLGILNRELKLKVFTL
>Csa004120
MGRGRVQLKRIENKISRQVTFSKRRAGLLKKAHEISVLCEADVALIVFSTKGKLFEYSSDSSMEKILEKYERYSYAE
RPLAPNGDSELQTSWCQEYPKLTARLEIVQKNLRHYLGEDLDPLNLRELQSLEQQLDTSLKRIRSRKEKDLQEEN
RQLANKVKENEKALVERGQCDVPNLVHNNQPIFGMTPPIPSLSFG
>Csa004592
MGRGRVQLKRIENKIIRQVTFSKRRSGLLKKAHEISVLCDAEVALIVFSTKGKLFEYSTDSCMEKILERYERYSYAER
RLVANDSQPNGNWTLEHAKLKARIEVLQKNHRHFMGEDLDSLSLKELQNIEQQLDSALKHIRARKNQLMHESI
TELKKKGKVLQEHNNILGKKIKEKEKSRAHNPQMEQQQHQNSNVIESSPLLLPQPFQSLSMSCPYPTHGLEENE
SAPNHERSDTLLPPWMLRHHLGD
>Csa013130
MGRGRVQLKRIENKINRQVTFSKRKAGLLKKAHEISVLCDAEVALIVFSHKGKLFEYSSDSSMEKILERYERYSFVG
RQQNAASESEFSYENWTLEYYRLKSKVELLQRNNSHYMGEDLDSLSVKELQNLEQQIDTALKHVRTRKVRNIEE
NNVQLAKQIKEKEKSVALAQQAEWEHQQQQGYNALSFLFPPPPHPSLNIG
>Csa014249
MGRGKVELKRIENPTSRQVTFSKRRNGLLKKAYELSVLCDAQVALLIFSPSGKAYQFSSHDMDGTLARYRTDVGL
PQSNHPHSRALFWKTEMDDMKRSISSMEARLRHFVGEDLEPLNVKELKQLERQMSVGIERIRSKKRRIIAEHIN
LLKRKYKGLQEEHSRLQKRLNQLKDVVVTNSSRNSDANPASALEIEFQVDGLLI
>Csa012493
MVRGKVEMKRIENSTSRQVTFSKRRNGLLKKAYELSVLCDAEVSVIIFSQKGRLYEFSSSDMQKTIERYRKHGKD
GQSNPFRSEGYMQQLKQEAEMTAKKIEQLEKSQQKLLGRGLDSCSFEEIREIERQLVLSLTRIRETKAQLFKEQKE
KLIEKKGEFLSEGNYKKDPEIIENVTREKNA

>Csa021114
MVRGKVEMKRIENATSRQVTFSKRRNGVLKKAYELSVLCDAEIAVIIFSQKGRLYEFASSEMPKIMDRYRKCTKD
AKNNDTKFDRQLLQQLRLEVESINKQMELMRLSHRKLLGYGLDNCSLDELEVLDAQLQRSLFQIRARKAQLYNE
QIQQLQEKNSLFEQVFSNSISLPKTLEE
>Csa014140
MVRGKTQMRLIENATSRQVTFSKRRNGLMKKAFELSVLCDAEVALIIFSPRGKLYEFASTSMQATIERYRKRAKA
KEALDPPFVNNIVQLEHLNHEEAASLIKKIEQLEVSKRKMLGEDLGSCSLDELQQLEHQLEKSVCKIRARKIEVFEE
QIKQLKQKEKVLQDENAKLLQKWESEGGDGGVNNEGGEKMLNYAESSSPSSEVETELLIGPPRRFLSIH
>Csa012879
MVRGKTQMKRIENATSRQVTFSKRRNGLLKKAFELSVLCDAEVALIIFSPRGKLYEFSNCSMNKTIDRYQNRTKD
LMSSNSTAIEDVQLEKEYDSFSMTKKLEHLEVCKRKLLGDGLDLCSIDELQQLERQLERSLSKIRSRKYQMLKDEI
MKLKEEEKMLLEENAALQIKVISESSKKQESNQRSESSNHEEIMDVETELFIGPPERRSNNNNNNNNNAFL
>Csa012099
MAKEKIQIRKIDNATARQVTFSKRRRGLFKKAKELSVLCDADVALIIFSATGKLFEYSSSSMKGIIERHNLHSKNLQK
LEQPSLELQLVENSNYTRLNKEIAEKTHQLRQMRGEELQTLNIEELQQLEKSLESGLSRVMEKKGERIMKEITDLQ
RKSAELMDENKRLKQQAEKMNGVRHLGVEPEILVVEDGQSSNSVTEVCVSNSNGPPQDLESSDTSLKLGLPYS
G
>Csa003859
MAREKIKIKKIDNLTARQVTFSKRRRGLIKKAEELSVLCDAEVALLVFSATGKFFEYSNSSIKDVIARYNLHSSNLGKL
EYPSIGLQVEDSNHVQLNKEVEDMNQQLRQMRGEDLQGLNLEDLKQLERKLEVGLTRVLHTKEKKIMREIDEL
ELKGARLMEENKMLKQQMLRLSNERLMAVLVDSSDVRVAAEEGLSSESAANVYSCNSGPPADDDSSDTSLKLG
SASLPKLRQKYFWRDGEGAK
>Csa017496
MTRQKIEIKKIDNIAARQVTFSKRRRGLFKKAHELATLCDADIALIVFSASGKLFDYSSSSTAGAKI
>Csa003446
MTRKKIQIKKIDNIAARQVAFSKRRKGLFKKAKELAILCDAEIGLLVFSASGKLFDYASSRFFFSHS
>Csa017887
MARGKIQIKRIENPTNRQVTYSKRRNGLFKKANELTVLCDAKVSIIMFSSTGKLHEYISPATSTKELFDQYQKTLGV
DLWITHYERMQDNLKKLKDINRNLRRQIRQRMGECMNDLSFEELRCLEQDMDNAVRIIRERKYRVISNQIETH
KKKLKSVGEIHKSLLQEFDIATEEDPHYGLVDNGGVGVGIGGGDYESIMGFSGAAHPRIFALRLQPNHTHNNHL
NNIHLHHPPPSDLTTYPLLE
>Csa011135
MGRGKIEIKRIENSSNRQVTYSKRRNGIIKKAKEITVLCDAQVSLVIFASSGKMHEYCSPSTPLVDILDKYHKQSGK
RLWDAKHERHLRGEDITSLNYKELMALEEALENGLTGVREKQSEFMKMMRTNERMMEEENKRLNYELYQKE
MVAMGDSVREMDIGYNQRMRDFNSQMPFAFRVQPIQPNLQERE
>Csa000681
MFQNQEEKMSDSPQRKMGRGKIEIKRIENTTNRQVTFCKRRNGLLKKAYEVMLKLLSSFSQAVAASMNMLTT
VLDLGHGSFKEGQDFGNLGCIENIVIYFFGEDLSVCWFSVWCIHRSGVTDGKIGHNMEWLMEG
>Csa017355
MSCYEEEDEESGVVGLRRSSSSSRTGRGKIEIKRIENTTNRQVTFCKRRNGLLKKAYELSVLCDAEVALIVFSSRGR
LYEYANNSVRATISRYKKAYSDPSTAMTVSEANTQFYQQESAKLRAQIGNLQNLNRHLLGESISSLSVKDLKSLEV
KLEKGISRIRSRKNELLFSEIEYMQKREIELHTNNQLIRAKIAETERSQQNTNASNNNGIATRRGEEGSMGTNLED
NNHHQYDSTNYFDPHHNHPISLQLV
>Csa021473

MGRGRVEIKKIENINSRQVTFSKRRNGLMKKAKELSVLCDAEVAIVVFSSTGRLYEFSSTSMEHTLSRYRGQGME
LDFPKETLDHPAQLPPSDSDAKSSEEEIGKLKLAYTQMRGQELDSLSFIDLQNLENQLREGIISIKDKKETLLLEQLQ
RCRSQLEEFQHRNNITLQESSPLQRSYFSDSKTASTNETEVKTEVEENDRSEISLHLGLSLDGQRKRKRSVEGAST
DTTCSQVELEGDALLANDELSRHFGM
>Csa020302
MGRGKIEIKRIENANSRQVTFSKRRAGLLKKAQELAILCDAEVAVIIFSNTGKLFEFSSSGMKHTLARYNKCVESS
DATVDVHKVELLGKDLTGLGFKELQNLEQQLNEGLLLVKEKKEQLLMEQLEQSRVQEQRAMLENETLRRQARR
CVNELRCLFPPVDCPLPAYLEYCSLEQKNIGIRSPDMACNSEIERGDSDTTLHLG
>Csa002117
MGRGKIVIRRIDNSTSRQVTFSKRRSGLLKKAKELAILCDADVGVIIFSSTSKLYEYSSTRIVSSVNCRTSIFVAETSFV
SVFHYSGNKLD
>Csa017500
MGRGKIVIRRIDNSTSRQVTFSKRRSGLLKKARELSILCDAEVGLIIFSSTGKLYDYSSSRSDLFLSLYNSPSLSLSLSLS
LSIYIYIYITFHNFIKWVFFLFLFFFHTIIIFHPNIIGSNN
>Csa012111
MGRGKIVIRRIDNSASRQVTFSKRRKGLIKKAKELSILCDAEVGLIIFSSSGKHYEFASSSMHSIIEKYNRRKEEDELL
LNPISDVKLWQKEVTTLRQQLHNLQENNRKLMGEQLYGLSMKDLNNLENQLEFSLQSIRIKKEQLLNDEIKELN
RKVYGHDSRSEMNLATGNALIPYGIIAAAPIAGDALCVPIHLQLSPREQQA
>Csa015983
MGRVKLQIKRIENTTNRQVTFSKRRNGLIKKAYELSILCDIDIALIMFSPSGRLSQFSGRRRIEDVLARYINLPDHD
RGSPTSSNSNVEELQQEVGTLRHELQLAEQQLRLFEPDFLSFTSNGEINSCEKNLLDTLARITQRKKDLLSTHLSPY
EPPNGIQIYLDQQDGIPTSFESDVGHWLPESNGQNNPNQICVASESSSIPQSGQYPTATVYDQVVSQAAAGTN
INVGVGVGVGVGGYDIANANDDGFSPWHHNYTTTQLLSSFIPQTSFDVVKNEIGEACMNTMVPQQQVDSIS
NGNQMPPSDGSANYDNVKLSQLNVD
>Csa008449
MGRKKIEVKLIEDRCNRHVTFCKRRSGLLKKAKELSVLCDVQVGIILFTNRGRLYEFSSGNRWTFLQSNVCFLLFL
FLLTVFTNKMSLVFGDLENRRNFVSDAFLAL
>Csa000939
MGRVKLKIKRLENTNGRQSTYSKRKNGIMKKAKELSILCDVDIILLMFSPTGKPNLSCEKRSFEEVIARFAQQTPQ
ERTKRKMESIDSLRKTFKKLDHDVNIDDLLGTSSQTIEDLTGQAKLLRTQLSEVHQRLSCWRNPDKINNVDHLSQ
MEDSMRETLNQVRLHKDNLQKHPPVPLEFTNQDGMHLTFDMSVEQQIQQLQHFSWIPNDSQNIVLHDDPN
FVLHRDAECSASSSFTSYPGYFGTGRSPEISNSGQENGVLPELSRTEALRPQLGGQNSYMSYNVNFFNDPTFQP
AAEMNLPINPVDYHVNGNLDTTQHNWASSSGPCAVSLLDDRLFP
>Csa004560
MKKARELAILCDIDIVLLMFSPSGKPALYEGERSNIEEVITKFAELTPQERAKRKMESLEVLKKTFKKLDHDVNIDD
FVGSSSQDFEELTNEASLLRDQIGETHKRLSYWRNPDSINNVDQLQQMEDLLRESLNQTRLHKENLRRHQLLS
QDFTGQYSCAGMSLPLLMEEMQGTQPLLWLANYGTQQIPLPNEPSFLQPGDVECSFPSYPSFFNPGKQIEAGI
SGQVDSMPQGDGALNELSGTSCSTLQLSDQYPYPTCDGSNFQDEKRLKMEMEMNLHAACVDTQLNDSSQID
SYQISPVIGGGNRLKKCSS
>Csa021069
MRKSRGRQKVEMVKMPNESNLQVTFSKRRSGLFKKASELCTLCGAEIAIIVFSPGKKVFSFGHPCVEALIERFVT
RNPPPSSGTLQLIEAHRNANVRELNAQLTQVLNQLEMERKRGEELNKLRKASQAQCWWELPIEEMEMHQLE
QLKASLDELKKNVTQQADRILIQTSSNANPPTQLIFPTQIPTQTSTTTNGPNQQPGLFVVDPKNIISHLPFNYGSY
GSRGFF

>Csa017317
MGRRKIEMKMVKDRGSRQVTFSKRRNGLFKKATDLATLCGLEIAIVVFSPGGKAFSFGNPNVEEVVDRYLGCE
WKANGNPGVRERGMLEKENEELLDLVKQLQMEKKKGEIMEKEMKSRGELMKIEDMDLNELLKLKESLEKLRK
NVKIEESELEASSSLLLLANEKPVPGDG
>Csa007119
MKKSSGRRKIEIKRLDKNTTRQVTFSKRRVGLFNKAAELSLLCGAEIAILLFSSRGKVYTFGHPNVDALLDRFLTGN
FLPPKPAEAYLPLPELNLDLCKAEAEFEIEKKRAVERLRNSERFWWDEALERMRMDELKSFRSSLLQLRANVAGR
LEKIRAMRMEDPPVTPSWSIVLGGENQCAASFHVGENQSFAMDWEEEIWG
>Csa020265
MKKSLGRQKIEIKKLNVKSRRQVTFSKRRAGLFNKAAELSILSGAEIAILVFSSTDKIYTFGHPNVDFLIDRFLTSNF
VPPKPVEAYLPLEELNRDLKDVTAEFETEKRRAERMRKTGGFWWDEAMECMGIEDLKRFRSSLMELRGKVAE
RVEELAAVRNQGFLTTSPSFHHLSVATEIDDLFYFSI
>Csa017909
MEKLNTIPMKKTLGRQKIEIKKLEKKSSKQVTFSKRRAGLFKKAGELSVLCGAEVAIIVFSPNDKLFCFGHPDVDVL
LDRYLTGNLSPPKPAESYIPVAEFNRDFADCALEFEAEKKRAAELIRAAEDSRKNGGFWWQEAVEGLRLEELKDF
RSALMDLRAKVAERVEKLTAVRIGGPLLPALPPPPQPMTSSSFHLIGNHQELPPPTAFHLAGNQRVATGLGFF
>Csa014962
MGRGILSLKLIPNPKSRRTTFLKRKKSLIKKAYELSTLCDVQTCLFIASDCDPSTHFETWPPNHHQIHQMIRSYKSH
SFTKPNSSYDLNRFFSDRKNKILTNTSKLLHNVVDHQSEHQLMELLDALDSKIRVANDMIEFMEADYDHLIDQAI
GMDTPPSQTEDEETTQFNVSDLFNEPDEFEEYNVEGFQSLLEDKFLETLVQNNPIPDFDFDFDY
>Csa017249
MGRGKLSMKLISNEKSRKTTFHKRKASLLRKAYELSTLCDVRVCVVVHGPNQSNDQSPLQLHTWPPSADEVN
NMIASYKTNCLYKRVRKSFGLIDFFSERKKKVETDMSKLRKDVSEERFPSWDERLDHLLEDQLRVLMVELDSKIEI
AKKRIEIATENNYNVEEGTSVESSGQTLNANMKCKQVMGFDHEEESTDYGMFGISFYNDPTNGMIMENTQSY
SSMCHYGVPFGAQSVVPISYMQMQQLTAGAEDQMMMGYASTSQMALPNTASSQVINDPFDFYNSYEYLMK
PNNF
>Csa002566
MTRKKVKLVWIASDNARKASFKKRRLGLLKKVSELTTLCGVYAFAVVTGPDEDHPVIWPSLSAAQHLYRRFHSLP
EVERQKKMTNQETYLKERTTKTQDLLKKHIKKNQELELDLLMHQLHQGRQIYQLTNGELLGLFWMIEERIRDCR
KRIEYHHQVHRLPPPPGLVTSNPALLETENNEMDLVDNGRNLMDQWFIDMVMNTNDKTGGSSSSMAGELG
FVQSEGNGVDMMTNGGGNSMMEASEIGGTGTIIVEGDGEENNLLSEWNFGGNDDCGMSEIEKLVNDIGGV
GHAELNASSMDLSHAQADFGIDCNVGGPMGSLFTDGVDVNDDGEMMLSGLFENEITENENVNQNGNDQN
NEEEDEEVEDEDDEDDILSKEWSNNFSSP
>Csa001552
MTRKKVKLAYIANDSARKATYKKRKRGLMKKVSELSTLCGIEACAIIFSPYDSQPELWPSPIGVQRVLSQFKKMPE
MEQSKKMVNQETFLRQRIAKANEQLKKMRKDNREKEITRLMFQSLTAAKGLHGLNMLDLNDLGWLIDQNLK
DITIRIDSLKIKPSSSQPQAQVQAQAQAPPTQPQTAAWLMELVSPQDQMGFVGDDMLLPFGDQTYNHNNA
MWSNAFFP
>Csa007130
MTRKKVKLAYITNDASRKATFKKRKKGLLKKLAELTTLCGIEACAIIFNPSNSQPDLWPSTLGLQKVLSKFKSLPEM
EQCKKMVNQETFLRDRIAKAADQLKKLQRENREKEITRVMFQSLVAGATPPLDLNVIDLNDLGWLVDQKMAD
IGKRMELLTVNRSSRVATNEPSWFMEMVNQGANDEDHMGFNIGDDVIQLPSFGEDDNHGTFWSNNNVIFP
>Csa025232
MGRGRVELKRIENKINRQVTFSKRRNGLLKKAYELSVLCDAEVALIIFSTRGKLYEFGSA

GTSKTLERYQRCCFSPQHNFAERETQNWFQEISKLKAKYESLCRTHRHLLGEDLGPLSVK
ELQNLEKQLEAALSQARQRKTQIMIEQMENLRKKERQLGILNRELKLKVFTL
>Csa026408
MGRGKVELKRIENPTSRQVTFSKRRNGLLKKAYELSVLCDAQVALLIFSPSGKAYQFSSH
DMDGTLARYRTDVGLPQSNHPHSRALFWKTEMDDMKRSISSMEARLRHFVGEDLEPLNVKELKQLERQMSV
GIERIRSKKRRIIAEHINLLKRKYKGLQEEHSRLQKRLNQLKDVVVTNS
SRNSDANPASALEIDFQSQVVYALDVRFRN
>Csa025231
MVRGKTQMRLIENATSRQVTFSKRRNGLMKKAFELSVLCDAEVALIIFSPRGKLYEFAST
SMQATIERYRKRAKAKEALDPPFVNNIVQLEHLNHEEAASLIKKIEQLEVSKRKMLGEDL
GSCSLDELQQLEHQLEKSVCKIRARKIEVFEEQIKQLKQKEKVLQDENAKLLQKWESEGG
DGGVNNEGGEKMLNYAESSSPSSEVETELLIGPPRRFLSIH
>CsaV3_4G010090
MGRGRVELKRIENKINRQVTFAKRRNGLLKKAYELSVLCDAEVALIIFSNRGKLYEFCSTSNMLKTLERYQKCSYG
AVEVTKPAKELESSYREYLKLKSR
FESLQRTQRNLLGEDLGPLNSKELEQLERQLVSSLKQVRSTKTQYMLDQLSDLQNKEQMLIETNRALQIKLEEISS
RNNIRLTWDGGDQSMSYGPQNAQT
QGFFQPLECNPTLQIGYTSAVSDQITSTTTPTHAQQVNGFLPGWML
>CsaV3_6G008200
MGRGRVELKRIENKINRQVTFAKRRNGLLKKAYELSVLCDAEVALIIFSNRGKLYEFCSSSSMLKTLERYQKCNYG
APEPNVSTREALELSSQQEYLKLK
ARYEALQRSQRNLLGEDLGPLSSKELESLERQLDMSLKQIRSTRTQYMLDQLTDLQRKEHLLNEANKTLKQRLVE
GYQVNALQLNQSADDMMYGRQQAQP
PGDAFFHPLDCEPTLQIGYQPDPITVVTAGPSMNNFLPGWLP
>CsaV3_1G006210
MGRGRVELKRIENKINRQVTFAKRRNGLLKKAYELSVLCDAELALIIFSNRGKLFEFCSGSSMTKTLEKYRRCSYGI
PNATHQVSVNQPQSFDDYLNLKA
TVEFMQQSQRNLLGEDLGPLNAKELEQLEHQLETSLERIRSTKTQSLLEQLTELQRKEQMLVEDNRGLKKKLEES
SAQVAVAAAGAWGWEDGAGGHNMEY
PSRGVASQSDAFFHPIVQPTPTLQIGYSSIGSMGMNHIGSPSQNANNNAFHLGWMI*
>CsaV3_6G033790
MGRGRVELKKIENKINRQVTFTKRRNGLLKKAYELSILCDAEVALIIFSARGKLYEFSSSPSIAKTLERYERHSYGALE
ASLPPKDTERWYQEYLKLKAE
VEALQYSQRRFLGEELDDLETKELDQLEIQLEMSLKQIRSTKRQTMFDQLSDLQKKEDKLLETNQALRKKLEESS
AAIHHTSWDSSEPNNLQYCRQPEAF
LQLNNNIIALENSYNPTEVTNEENVVNSGADGNGLSSHWMLL*
>CsaV3_6G006010
MGRGRVELKRIENKINRQVTFSKRRNGLLKKAYELSVLCDAEVALIIFSTRGKLYEFGSAGTSKTLERYQRCCFSPQ
HNFAERETQNWFQEISKLKAKYE
SLCRTHRHLLGEDLGPLSVKELQNLEKQLEAALSQARQRKTQIMIEQMENLRKKERQLGILNRELKLKLEAEGQN
VRGIESFWSCGSGSGHPNNNFPLHH
PLQPDPIDCQHQPLLQIGYQNYFSEEGPSHVQKTMTCETNFIQGWVI*
>CsaV3_4G010080
MGRGRVQLKRIENKISRQVTFSKRRAGLLKKAHEISVLCEADVALIVFSTKGKLFEYSSDSSMEKILEKYERYSYAE

RPLAPNGDSELQTSWCQEYPKLT
ARLEIVQKNLRHYLGEDLDPLNLRELQSLEQQLDTSLKRIRSRKNQLMQESISILHKKEKDLQEENRQLANKVKE
NEKALVERGQCDVPNLVHNNQPIFG
MTPPIPSLSFGANLNGRGSRGSDEDETRPTSINNIQIPAWMLRHQPHRVVFAVAGRQAQVVATAQLTGTIIVTN
KKAMGMGAEKREDNYKRLVDIENGDA
KNIAILRIQLPLRFVSPNRLPIPSSQFFQGECKFIRIMAESVGSRNMDKKLIQIDISSDTVCPWCFVGKKNLDKAIS
ASQDQYDFELNWHPFQLNPTAPK
EGVVKTEYYRSKFGIQSEQMEARMAEVFRGLGLDYDTSGLTGNTLESHKLIYLAGQQGLGKQHDLVEELCLGYF
TQGKYIGDRDFLLECARKAGVEGAAE
FLETADNGVKEVKEELEKYSGKISGVPFYVINGKHKLSGAQPPEVFLRAFQVAGK*
>CsaV3_1G006220
MGRGRVQLKRIENKIIRQVTFSKRRSGLLKKAHEISVLCDAEVALIVFSTKGKLFEYSTDSCEMNMSQNLNFHFS
MEKILERYERYSYAERRLVANDSQP
NGNWTLEHAKLKARIEVLQKNHRHFMGEDLDSLSLKELQNIEQQLDSALKHIRARKNQLMHESITELKKKGKVL
QEHNNILGKKIKEKEKSRAHNPQMEQ
QQHQNSNVIESSPLLLPQPFQSLSMSCPYPTHGLEENESAPNHERSDTLLPPWMLRHHLGD*
>CsaV3_6G033800
MGRGRVQLKRIENKINRQVTFSKRKAGLLKKAHEISVLCDAEVALIVFSHKGKLFEYSSDSSMEKILERYERYSFVG
RQQNAASESEFSYENWTLEYYRL
KSKVELLQRNNSHYMGEDLDSLSVKELQNLEQQIDTALKHVRTRKNQLMFESITDLQKKVRNIEENNVQLAKQI
KEKEKSVALAQQAEWEHQQQQGYNAL
SFLFPPPPHPSLNIGLASFDGHQ*
>CsaV3_1G009750
MGRGKVELKRIENPTSRQVTFSKRRNGLLKKAYELSVLCDAQVALLIFSPSGKAYQFSSHDMDGTLARYRTDVGL
PQSNHPHSRALFWKTEMDDMKRSIS
SMEARLRHFVGEDLEPLNVKELKQLERQMSVGIERIRSKKRRIIAEHINLLKRKYKGLQEEHSRLQKRLNQLKDVV
VTNSSRNSDANPASALEIEFQVDG
LLI*
>CsaV3_5G005600
MLLLVLRVRLRNGDRFPFLPLLGFLLSSSHFHTIIFSFFFFFFFTLHSSMILYKTPWDKFGKNDMVRGKVEMKRIE
NSTSRQVTFSKRRNGLLKKAYELS
VLCDAEVSVIIFSQKGRLYEFSSSDMQKTIERYRKHGKDGQSNPFRSEGYMQQLKQEAEMTAKKIEQLEKSQQK
LLGRGLDSCSFEEIREIERQLVLSLT
RIRETKAQLFKEQKEKLIEKGKLLLEENLKLSAKCGTKPWQEEGVEGDGGINMMSNLCSQSTNSQASDHHMQT
DLFIGLSCS*
>CsaV3_3G016650
MVRGKVEMKRIENATSRQVTFSKRRNGVLKKAYELSVLCDAEIAVIIFSQKGRLYEFASSEMPKIMDRYRKCTKD
AKNNDTKFDRQLLQQLRLEVESINK
QMELMRLSHRKLLGYGLDNCSLDELEVLDAQLQRSLFQIRARKAQLYNEQIQQLQEKEKLLLEENRILSLKKIFEKI
GNGGWS*
>CsaV3_3G009400
MVRGKTQMRRIENATSRQVTFSKRRNGLLKKAFELSVLCDAELALIIFSSRGKLYEFSSSSMQATVGRYLRHTRSD
QSHHLDQPLPHQHDLQAVQNEAAS
LLKEIESVEVSKRKLLGESLGTSSYEELQQLEQQLERSLSHIRARKHEVYREQIEQLKEKEKHLTAENAKLAKKYDVE

EERQQSPTQIQLAEVSPNYGES
SSISDVETDLFIGPPKSRSK*
>CsaV3_6G006020
MVRGKTQMRLIENATSRQVTFSKRRNGLMKKAFELSVLCDAEVALIIFSPRGKLYEFASTSMQATIERYRKRAKA
KEALDPPFVNNIVQLEHLNHEEAAS
LIKKIEQLEVSKRKMLGEDLGSCSLDELQQLEHQLEKSVCKIRARKIEVFEEQIKQLKQKEKVLQDENAKLLQKWE
SEGGDGGVNNEGGEKMLNYAESSS
PSSEVETELLIGPPRRFLSIH*
>CsaV3_5G003360
MVRGKTQMKRIENATSRQVTFSKRRNGLLKKAFELSVLCDAEVALIIFSPRGKLYEFSNCSMNKTIDRYQNRTKD
LMSSNSTAIEDVQLEKEYDSFSMTK
KLEHLEVCKRKLLGDGLDLCSIDELQQLERQLERSLSKIRSRKYQMLKDEIMKLKEEEKMLLEENAALQIKVISESS
KKQESNQRSESSNHEEIMDVETE
LFIGPPERRSNNNNNNNNNAFL*
>CsaV3_6G045010
MAKEKIQIRKIDNATARQVTFSKRRRGLFKKAKELSVLCDADVALIIFSATGKLFEYSSSSMKGIIERHNLHSKNLQK
LEQPSLELQLVENSNYTRLNKE
IAEKTHQLRQMRGEELQTLNIEELQQLEKSLESGLSRVMEKKGERIMKEITDLQRKSAELMDENKRLKQQAEKM
NGVRHLGVEPEILVVEDGQSSNSVTE
VCVSNSNGPPQDLESSDTSLKLGLPYSG*
>CsaV3_2G030300
MAREKIKIKKIDNLTARQVTFSKRRRGLIKKAEELSVLCDAEVALLVFSATGKFFEYSNSSIKDVIARYNLHSSNLGKL
EYPSIGLQVEDSNHVQLNKEV
EDMNQQLRQMRGEDLQGLNLEDLKQLERKLEVGLTRVLHTKEKKIMREIDELELKGARLMEENKMLKQQMLR
LSNERLMAVLVDSSDVRVAAEEGLSSES
AANVYSCNSGPPADDDSSDTSLKLGPPCPN*
>CsaV3_4G014770
MTRQKIEIKKIDNIAARQVTFSKRRRGLFKKAHELATLCDADIALIVFSASGKLFDYSSSSMLDLLRRHNMLPELNS
ISQPPSQLEKSAHAKLTEEFAAK
TKELRHMKGEELQELGIEELKQLEKLLENGLNRVIETKDEKFLKEIVTVKEKESLLMKENQRLRNKLPFWCFL*
>CsaV3_7G006940
MTRKKIQIKKIDNIAARQVAFSKRRKGLFKKAKELAILCDAEIGLLVFSASGKLFDYASSSIQEILERHNSVHSENLP
NLNEPSVELQLESNIRAKLNEE
VEKKSHELRQMKGEELQGLGMEELKKLEKSLQGGLSRVAEIMDGKNTDLLSDIGRKVDLLIEENKRLNQLEVDK
LGEQIMQNIQGHSSESIGNNSTSSNN
PSQDYDSSDTSLKLGLPLLDF*
>CsaV3_5G040310
MARGKVQMKRIENPVHRQVTFCKRRAGLLKKAKELSVLCDAEIGLFIFSAHGKLYELATKGTMQGLIERYMKHT
NGNQPPDPPIHHQTLEVKEEIIRLKQ
EIEVLKGLRNALGGGGGEGIMTLDELDIFEKQLEIWICQVRSTKMKFMCEEIEALRNQEFILTAANKYLHDKMEA
ENISNTTNMEPVNITNCQYPLTIQD
ELFQLCT*
>CsaV3_6G052910
MGRGKIAIRRIENRTTRQVTFSKRRGGLFKKTHELSVLCDAQIALIVFSSNGKLFEYCTQTTCMDQIIRRYQIAIGS

RIPEHNMNDPEELERQVRSMKKE
TDDLQLSLQRYTADDFSTFHLRDLDDIENRLQNSLNRVRARKSELLQQQVDNLRRKEKILEDENEQIYHLIKEEQ
MAMEEQQQQQMAAAVMMQKRRSEED
DEEVMMRLRRRAAHDNHDNAADDQAQAAAVELQLQLQHDYHNHHHHHHHHHHDQASSCVLQLASVQQ
QLPSDPSPPPPPFLLPYRLQPFQPNLQDINLHS
STYE*
>CsaV3_3G045590
MYVYFSHKQQHQQQQERRRSRRRRRRRRKRKNMARGKIQIKRIENPTNRQVTYSKRRNGLFKKANELTVLCD
AKVSIIMFSSTGKLHEYISPATSTKELF
DQYQKTLGVDLWITHYERMQDNLKKLKDINRNLRRQIRQRMGECMNDLSFEELRCLEQDMDNAVRIIRERKY
RVISNQIETHKKKLKSVGEIHKSLLQEF
DIATEEDPHYGLVDNGGVGVGIGGGDYESIMGFSGAAHPRIFALRLQPNHTHNNHLNNIHLHHPPPSDLTTYPL
LE*
>CsaV3_4G028010
MGRGKIEIKRIENSSNRQVTYSKRRNGIIKKAKEITVLCDAQVSLVIFASSGKMHEYCSPSTPLVDILDKYHKQSGK
RLWDAKHENLSNEMDRVKKENDN
MQIELRHLRGEDITSLNYKELMALEEALENGLTGVREKQSEFMKMMRTNERMMEEENKRLNYELYQKEMVA
MGDSVREMDIGYNQRMRDFNSQMPFAFRV
QPIQPNLQERE*
>CsaV3_6G015770
MGRGKIEIKRIENTTNRQVTFCKRRNGLLKKAYELSVLCDAEVALIVFSTRGRLYEYANNSVRGTIERYKKAFADSS
NSGLSVAEANVQFYQQEATKLKR
QIREIQNSNRHILGEALSSLPLKELKSLEGRLERGISKVRAKKNETLFAEMEFMQKREMELQSHNNYLRTQIAEHE
RIQQQQQQQQQTNMMQRATYESVG
GQYDDENRSTYGAVGALMDSDSHYAPQDHLTALQLV*
>CsaV3_1G032920
MSCYEEEDEESGVVGLRRSSSSSRTGRGKIEIKRIENTTNRQVTFCKRRNGLLKKAYELSVLCDAEVALIVFSSRGR
LYEYANNSVRATISRYKKAYSDP
STAMTVSEANTQFYQQESAKLRAQIGNLQNLNRHLLGESISSLSVKDLKSLEVKLEKGISRIRSRKNELLFSEIEYM
QKREIELHTNNQLIRAKIAETER
SQQNTNASNNNGIATRRGEEGSMGTNLEDNNHHQYDSTNYFDPHHNHPISLQLV*
>CsaV3_6G051220
MFQNQEEKMSDSPQRKMGRGKIEIKRIENTTNRQVTFCKRRNGLLKKAYELSVLCDAEVALIVFSSRGRLYEYA
NNSVKATIDRYKKASSDSSNTGSTSE
ANTQFYQQEAAKLRVQIGNLQNSNRNMLGESLSSLTAKDLKGLETKLEKGISRIRSKKNELLFAEIEYMRKREIDL
HNNNQMLRAKIAESERNVNMMGGE
FELMQSHPYDPRDFFQVNGLQHNHQYPRQDNMALQLV*
>CsaV3_5G040370
MGRGKIEIKRIENTTNRQVTFCKRRNGLLKKAYELSVLCDAEVALIVFSSRGRLYEYSNNSIKTTIERYKKACSDSSA
TSSVTELNTQYYQQESAKLRQQ
IQMLQNSNRHLMGDSLSALTVKELKQLENRLERGITRIRSKKHEMLLAEIEYLQKREIELENENVCIRTKIAEVERV
QQANMVSGQELNAIQALANSRNF
FSPNIMEPAGPVSYSHQDKKMLHLG*
>CsaV3_4G028880

MFGPKEQNPLPPTPKSTMGRGRVEIKKIENINSRQVTFSKRRNGLMKKAKELSVLCDAEVAIVVFSSTGRLYEFS
STSMEHTLSRYRGQGMELDFPKETL
DHPAQLPPSDSDAKSSEEEIGKLKLAYTQMRGQELDSLSFIDLQNLENQLREGIISIKDKKETLLLEQLQRCRSQGE
VVISENETLRKQLEEFQHRNNIT
LQESSPLQRSYFSDSKTASTNETEVKTEVEENDRSEISLHLGLSLDGQRKRKRSVEGASTDTTCSQVELEGDALLA
NDELSRHFGM*
>CsaV3_3G031900
MGRGKIEIKRIENANSRQVTFSKRRAGLLKKAQELAILCDAEVAVIIFSNTGKLFEFSSSGMKHTLARYNKCVESS
DATVDVHKVEREHEEVDILREEIT
TLQMKQLQLLGKDLTGLGFKELQNLEQQLNEGLLLVKEKKEQLLMEQLEQSRVQEQRAMLENETLRRQVNELR
CLFPPVDCPLPAYLEYCSLEQKNIGIR
SPDMACNSEIERGDSDTTLHLGLPSHVYCKRKESERDTHSNDSGAQMGIL*
>CsaV3_3G048150
MGRGKIVIRRIDNSTSRQVTFSKRRSGLLKKAKELAILCDADVGVIIFSSTSKLYEYSSTSMKALIERYNKTKEENHQ
LGIPTSEVKYWQREAATLRQQL
QSLHENHRQMMGEELTGLSVKDLQNLENQLEISLRGVRMKKDQILMEEIQELNRKGNLIHHDNMELYKKVYG
TKDANGAHISSITNGLSVGEDAGIPINL
QLSQPQQQDNEAPERATKLGRLQLR*
>CsaV3_4G014700
MGRGKIVIRRIDNSTSRQVTFSKRRSGLLKKARELSILCDAEVGLIIFSSTGKLYDYSSSSIRSITDRYNKMKEEQNQ
LMNSVSELQFWKREAAALKQQL
HYLQECHRQLMGEELSGLSVKDLQNLESQLEMSLKGVRVKKEKTLSDEITELKQKGNHMHQENVELYKRLDMT
RKENAELQMKYQAYGPMEIDKTSSSSQ
QFTITNRYSMPALQLRQPQPQNNETPGIKLGLQLQ*
>CsaV3_6G044810
MGRGKIVIRRIDNSASRQVTFSKRRKGLIKKAKELSILCDAEVGLIIFSSSGKHYEFASSSMHSIIEKYNRRKEEDELL
LNPISDVKEVTTLRQQLHNLQ
ENNRKLMGEQLYGLSMKDLNNLENQLEFSLQSIRIKKEQLLNDEIKELNRKGILMHQENIELSNKVSLYCQENME
LHRKVYGHDSRSEMNLATGNALIPY
GIIAAAPIAGDALCVPIHLQLSPREQQA*
>CsaV3_4G030750
MGRVKLQIKRIENTTNRQVTFSKRRNGLIKKAYELSILCDIDIALIMFSPSGRLSQFSGRRRIEDVLARYINLPDHD
RGSVVQNKEFLLGTLNNLKTEND
IAQQLTNPTSSNSNVEELQQEVGTLRHELQLAEQQLRLFEPDFLSFTSNGEINSCEKNLLDTLARITQRKKDLLST
HLSPYEPPNGIQIYLDQQDGIPTS
FESDVGHWLPESNGQNNPNQICVASESSSIPQSGQYPTATVYDQVVSQAAAGTNINVGVGVGVGVGGYDIAN
ANDDGFSPWHHNYTTTQLLSSFIPQTSF
DVVKNEIGEACMNTMVPQQQVDSISNGNQMPPSDGSANYDNVKLSQLNVD*
>CsaV3_6G008210
MGRKKIEVKLIEDRCNRHVTFCKRRSGLLKKAKELSVLCDVQVGIILFTNRGRLYEFSSGNSLLNIIMRYQSHLQG
RNESPIDNDLQGTSESLIDNDAKD
HVSDETILVSLKKLLQTIQSQVEEPNFKKLDITQMVQLENQLESTLDKIKSQRIEAMIENDDCWTYDMDMAMG
MINSPPFN*
>CsaV3_5G032860

MGRVKLKIKRLENTNGRQSTYSKRKNGIMKKAKELSILCDVDIILLMFSPTGKPNLSCEKRSFEEVIARFAQQTPQ
ERTKRKMESIDSLRKTFKKLDHDV
NIDDLLGTSSQTIEDLTGQAKLLRTQLSEVHQRLSCWRNPDKINNVDHLSQMEDSMRETLNQVRLHKDNLQK
HPPVPLEFTNQDGMHLTFDMSVEQQIQQ
LQHFSWIPNDSQNIVLHDDPNFVLHRDAECSASSSFTSYPGYFGTGRSPEISNSGQENGVLPELSRTEALRPQLG
GQNSYMSYNVNFFNDPTFQPAAEMN
LPINPVDYHVNGNLDTTQHNWASSSGPCAVSLLDDRLFP*
>CsaV3_1G005580
MGRVKLKIKKLESTGSRQVTYSKRRNGIMKKARELAILCDIDIVLLMFSPSGKPALYEGERSNIEEVITKFAELTPQE
RAKRKMESLEVLKKTFKKLDHD
VNIDDFVGSSSQDFEELTNEASLLRDQIGETHKRLSYWRNPDSINNVDQLQQMEDLLRESLNQTRLHKENLRR
HQLLSQDFTGQYSCAGMSLPLLMEEMQ
GTQPLLWLANYGTQQIPLPNEPSFLQPGDVECSFPSYPSFFNPGKQIEAGISGQVDSMPQGDGALNELSGTSCS
TLQLSDQYPYPTCDGSNFQDEKRLKM
EMEMNLHAACVDTQLNDRLELSRSLYDDNQHPWASIPGPCSIPMYQSNEYHHQPN*
>CsaV3_3G016620
MGRGKVVLERIENRVNRQVTFSKRRNGLLKKASELSVLCDVDVALIIFSTRGKLFEFGSTDMNKILERYHQQCYT
SGSTTNLDESDVQIEEVSKLRAKYE
SLQRSHRNFLGEELEPLTLKELHNLEKQLDKTLSQARQRKAEIMLQKLADLRKMEQDLGDQNTQLKSKLEKDQE
QEGGEEDPKNYEVVRADDPNMINTTR
YYEAQEEEEECRGVIDGGSNLIPDWLL*
>CsaV3_4G000010
MRKSRGRQKVEMVKMPNESNLQVTFSKRRSGLFKKASELCTLCGAEIAIIVFSPGKKVFSFGHPCVEALIERFVT
RNPPPSSGTLQLIEAHRNANVRELN
AQLTQVLNQLEMERKRGEELNKLRKASQAQCWWELPIEEMEMHQLEQLKASLDELKKNVTQQADRILIQTSS
NANPPTQLIFPTQIPTQTSTTTNGPNQQ
PGLFVVDPKNIISHLPFNYGSYGSRGFF*
>CsaV3_1G032570
MGRRKIEMKMVKDRGSRQVTFSKRRNGLFKKATDLATLCGLEIAIVVFSPGGKAFSFGNPNVEEVVDRYLGCE
WKANGNPGVRERGMLEKENEELLDLVK
QLQMEKKKGEIMEKEMKSRGELMKIEDMDLNELLKLKESLEKLRKNVKIEESELEASSSLLLLANEKPVPGDG*
>CsaV3_1G015790
MDLFTADHRIPTSDNFPQHVAPFPDPTDLLYAAPSSVFPPTDIINHLSNPPPPPQKLRPIRCNGRSPAGSQAENIF
DGSLRSFQCVSSSPEGGFSGDQLC
VANIDPCQYFNSSAKDEKPEVKHNGSFGDIIANDYFSEEETKNGGSGAAIAAENLSRSREEPQLDDDSCSTSDG
GDAVFSSKKHLSHKRKRTRRSLEHFV
EKLVMKVMDKQEEMHRQLIDMIEKKENERTVREEAWKQREIERIKRDEELRAQETSRSLAIISLIQNLLGHEIQIS
RPAENQCAEDDGGESSIQKELKCD
PSGRRWPQAEVQSLISLRTSLEHKFRATGSKGSIWEEISIEMQKMGYKRSAKKCKEKWENMNKYFKRTVVTGK
ASIANGKTCPYFQELDILYRNGVVNTG
AVFDSTNTENNSNAERSIDPFHEDAFVEGEREHIKQEEALDMKSSGRRKIEIKRLDKNTTRQVTFSKRRVGLFNK
AAELSLLCGAEIAILLFSSRGKVYT
FGHPNVDALLDRFLTGNFLPPKPAEAYLPLPELNLDLCKAEAEFEIEKKRAVERLRNSERFWWDEALERMRMDE
LKSFRSSLLQLRANVAGRLEKIRAMR

MEDPPVTPSWSIVLGGENQCAASFHVGENQSFAMDWEEEIWG*
>CsaV3_2G016620
MKKSLGRQKIEIKKLNVKSRRQVTFSKRRAGLFNKAAELSILSGAEIAILVFSSTDKIYTFGHPNVDFLIDRFLTSNF
VPPKPVEAYLPLEELNRDLKDV
TAEFETEKRRAERMRKTGGFWWDEAMECMGIEDLKRFRSSLMELRGKVAERVEELAAVRNQGFLTTSPSFHH
LSVATEIDDLFYFSI*
>CsaV3_3G045410
MEKLNTIPMKKTLGRQKIEIKKLEKKSSKQVTFSKRRAGLFKKAGELSVLCGAEVAIIVFSPNDKLFCFGHPDVDVL
LDRYLTGNLSPPKPAESYIPVAE
FNRDFADCALEFEAEKKRAAELIRAAEDSRKNGGFWWQEAVEGLRLEELKDFRSALMDLRAKVAERVEKLTAV
RIGGPLLPALPPPPQPMTSSSFHLIGN
HQELPPPTAFHLAGNQRVATGLGFF*
>CsaV3_UNG063480
MKKSSGRRKIEIKRLDKNTTRQVTFSKRRVGLFNKAAELSLLCGAEIAILLFSSRGKVYTFGHPNVDALLDRFLTGN
FLPPKPAEAYLPLPELNLDLCKA
EAEFEIEKKRAVERLRNSERFWWDEALERMRMDELKSFRSSLLQLRANVAGRLEKIRAMRMEDPPVTPSWSIV
LGGENQCAASFHVVKISRLRWIGKRRF
GVSAQHIAPEAATCHIHTKI*
>CsaV3_6G051590
MDSSAPNAPVAGNKKQTKGRQKIEMKKIVNEDDRLITFSKRRSGIYKKASELATLCGAEVGVVVFSPAGKPFSFA
HPCIETIANKFLNGNKNNKGNNDDN
NNNGDSSSSNNNNNNSNNNNNSNAAHPLVEAHRRVRINELNQQHNQLLSQLDAEKEKGKALEKLKRVRGN
GRGLL*
>CsaV3_3G020270
MITPVFSVLLIPSTIAQNLLSHWSFLRFALLRRAMGRGILSLKLIPNPKSRRTTFLKRKKSLIKKAYELSTLCDVQTCL
FIASDCDPSTHFETWPPNHHQ
IHQMIRSYKSHSFTKPNSSYDLNRFFSDRKNKILTNTSKLLHNVVDHQSEHQLMELLDALDSKIRVANDMIEFM
EADYDHLIDQAIGMDTPPSQTEDEET
TQFNVSDLFNEPDEFEEYNVEGFQSLLEDKFLETLVQNNPIPDFDFDFDY*
>CsaV3_2G019470
MGRGKLSMKLISNEKSRKTTFHKRKASLLRKAYELSTLCDVRVCVVVHGPNQSNDQSPLQLHTWPPSADEVN
NMIASYKTNCLYKRVRKSFGLIDFFSER
KKKVETDMSKLRKDVSEERFPSWDERLDHLLEDQLRVLMVELDSKIEIAKKRIEIATENNYNVEEGTSVESSGQT
LNANMKCKQVMGFDHEEESHQWSKH
KA*
>CsaV3_3G038110
MTRKKVKLVWIASDNARKASFKKRRLGLLKKVSELTTLCGVYAFAVVTGPDEDHPVIWPSLSAAQHLYRRFHSLP
EVERQKKMTNQETYLKERTTKTQDL
LKKHIKKNQELELDLLMHQLHQGRQIYQLTNGELLGLFWMIEERIRDCRKRIEYHHQVHRLPPPPGLVTSNPALL
ETENNEMDLVDNGRNLMDQWFIDMV
MNTNDKTGGSSSSMAGELGFVQSEGNGVDMMTNGGGNSMMEASEIGGTGTIIVEGDGEENNLLSEWNFG
GNDDCGMSEIEKLVNDIGGVGHAELNASSMD
LSHAQADFGIDCNVGGPMGSLFTDGVDVNDDGEMMLSGLFENEITENENVNQNGNDQNNEEEDEEVEDE
DDEDDILSKEWSNNFSSP*

>CsaV3_1G038060
MTRKKVKLAYIANDSARKATYKKRKRGLMKKVSELSTLCGIEACAIIFSPYDSQPELWPSPIGVQRVLSQFKKMPE
MEQSKKMVNQETFLRQRIAKANEQ
LKKMRKDNREKEITRLMFQSLTAAKGLHGLNMLDLNDLGWLIDQNLKDITIRIDSLKIKPSSSQPQAQVQAQAQ
APPTQPQTAAWLMELVSPQDQMGFVG
DDMLLPFGDQTYNHNNAMWSNAFFP*
>CsaV3_1G017300
MTRKKVKLAYITNDASRKATFKKRKKGLLKKLAELTTLCGIEACAIIFNPSNSQPDLWPSTLGLQKVLSKFKSLPEM
EQCKKMVNQETFLRDRIAKAADQ
LKKLQRENREKEITRVMFQSLVAGATPPLDLNVIDLNDLGWLVDQKMADIGKRMELLTVNRSSRVATNEPSWF
MEMVNQGANDEDHMGFNIGDDVIQLPS
FGEDDNHDSSINV*

“*” represents a stop codon
