# Supplementary material for: Genome-Wide Reidentification and Expression Analysis of MADS-Box Gene Family in Cucumber
Source: Int J Mol Sci. 2025 Apr 17;26(8):3800. doi: 10.3390/ijms26083800 (PMC12027882; doi:10.3390/ijms26083800)
Supplement: Supplementary file 1 [file ijms-26-03800-s001.zip › Figure S1.pptx]

## Slide 1
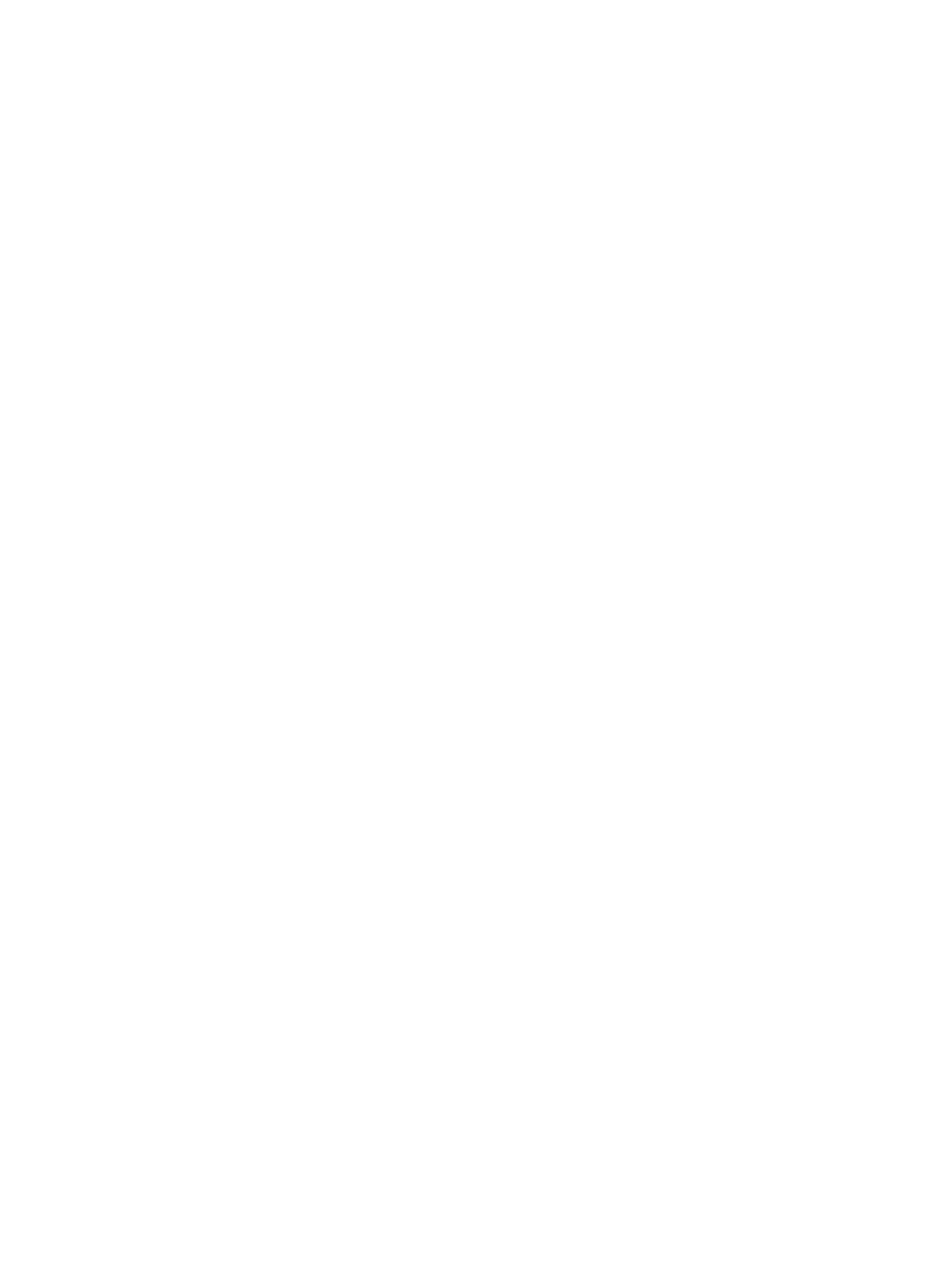

#

## Slide 2
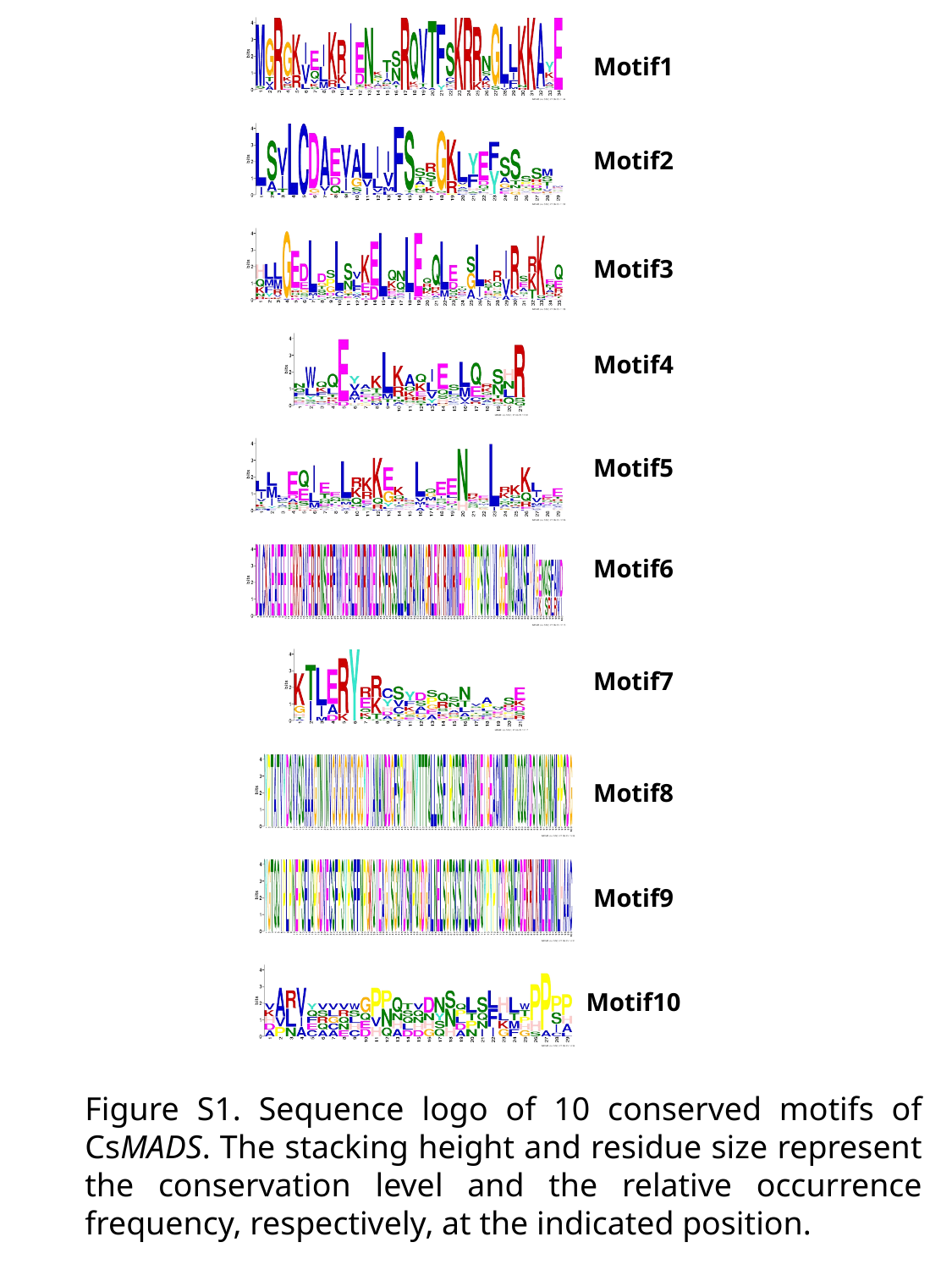

Motif1
Motif2
Motif3
Motif4
Motif5
Motif6
Motif7
Motif8
Motif9
Motif10
Figure S1. Sequence logo of 10 conserved motifs of CsMADS. The stacking height and residue size represent the conservation level and the relative occurrence frequency, respectively, at the indicated position.
